# Bayesian calibration of a stochastic, multiscale agent-based model for predicting *in vitro* tumor growth

**DOI:** 10.1371/journal.pcbi.1008845

**Published:** 2021-11-29

**Authors:** Ernesto A. B. F. Lima, Danial Faghihi, Russell Philley, Jianchen Yang, John Virostko, Caleb M. Phillips, Thomas E. Yankeelov

**Affiliations:** 1 Oden Institute for Computational Engineering and Sciences, The University of Texas at Austin, Austin, Texas, United States of America; 2 Texas Advanced Computing Center, The University of Texas at Austin, Austin, Texas, United States of America; 3 Department of Mechanical and Aerospace Engineering, University at Buffalo, Buffalo, New York, United States of America; 4 Department of Biomedical Engineering, The University of Texas at Austin, Austin, Texas, United States of America; 5 Department of Diagnostic Medicine, The University of Texas at Austin, Austin, Texas, United States of America; 6 Department of Oncology, The University of Texas at Austin, Austin, Texas, United States of America; 7 Livestrong Cancer Institutes, Dell Medical School, The University of Texas at Austin, Austin, Texas, United States of America; 8 Department of Imaging Physics, The University of Texas MD Anderson Cancer Center, Houston, Texas, United States of America; University of Southern California, UNITED STATES

## Abstract

Hybrid multiscale agent-based models (ABMs) are unique in their ability to simulate individual cell interactions and microenvironmental dynamics. Unfortunately, the high computational cost of modeling individual cells, the inherent stochasticity of cell dynamics, and numerous model parameters are fundamental limitations of applying such models to predict tumor dynamics. To overcome these challenges, we have developed a coarse-grained two-scale ABM (cgABM) with a reduced parameter space that allows for an accurate and efficient calibration using a set of time-resolved microscopy measurements of cancer cells grown with different initial conditions. The multiscale model consists of a reaction-diffusion type model capturing the spatio-temporal evolution of glucose and growth factors in the tumor microenvironment (at tissue scale), coupled with a lattice-free ABM to simulate individual cell dynamics (at cellular scale). The experimental data consists of BT474 human breast carcinoma cells initialized with different glucose concentrations and tumor cell confluences. The confluence of live and dead cells was measured every three hours over four days. Given this model, we perform a time-dependent global sensitivity analysis to identify the relative importance of the model parameters. The subsequent cgABM is calibrated within a Bayesian framework to the experimental data to estimate model parameters, which are then used to predict the temporal evolution of the living and dead cell populations. To this end, a moment-based Bayesian inference is proposed to account for the stochasticity of the cgABM while quantifying uncertainties due to limited temporal observational data. The cgABM reduces the computational time of ABM simulations by 93% to 97% while staying within a 3% difference in prediction compared to ABM. Additionally, the cgABM can reliably predict the temporal evolution of breast cancer cells observed by the microscopy data with an average error and standard deviation for live and dead cells being 7.61±2.01 and 5.78±1.13, respectively.

## Author summary

The calibration of agent-based models of tumor cell growth to experimental data remains a challenge in computational oncology. Besides the computational cost of modeling thousands of agents, the model’s intrinsic stochasticity demands numerous realizations of the simulations to accurately represent the statistical features of the model predictions. We developed a hybrid, multiscale, coarse-grain, agent-based model that captures the growth and decline of human breast carcinoma cells under different initial conditions. We determined the effects of coarse-graining the ABM on the multiscale model output and the number of repetitions necessary to capture the stochastic transitions present in the model. We identified the most influential parameters on the model prediction through a sensitivity analysis and selected which parameters can be fixed and which ones should be calibrated. Using Bayesian calibration, we show that the model can accurately represent the experimental data. The validation step indicates that our model can reliably predict the *in vitro* temporal data, depending on the choice of the training (calibration data) sets.

## Introduction

Tumor growth and treatment response are governed by the complex interplay of numerous phenomena occurring at various spatial and temporal scales. Hybrid multiscale models of tumor development consist of discrete models of individual cell interactions and their phenotypic transitions coupled to continuum models of microenvironmental evolution. These models allow investigation of the complex mechanisms of tumor initiation and growth at the interface of cellular, microenvironmental, and tissue scales events. More specifically, hybrid multiscale agent-based models (ABMs) consist of a continuum model capturing the spatiotemporal evolution of nutrients and growth factors in the tumor microenvironment. At the cellular scale, agent-based models simulate individual cell division and growth, cell-cell and cell-microenvironment interactions, and phenotypic switches that follow a user-defined set of probabilistic rules. The discrete agent-based and continuum models are coupled such that cellular dynamics influence the continuum model through nutrient consumption, while the concentration of nutrient impacts the decision-making process of the individual agents in the cell-scale model [1–19]. Compared to models governed by differential equations (see, e.g., [20–24]), the primary benefit of combining multiple models in ABMs is the ability to simulate coupled, multiscale processes and mechanisms responsible for tumor growth and treatment response [25]. This provides an opportunity to computationally test a range of hypotheses on the underlying biological phenomena driving cancer development. In this regard, hybrid ABMs have become powerful computational tools to study the complex and multiscale processes of tumor development including, for example, proliferation [14, 26], migration [11, 27], invasion [3, 5], angiogenesis [28, 29], treatment effects [30], along with mechanical [3, 31, 32] and biochemical [11, 26] cues. For example, Macklin *et al*. [1, 26] used an agent-based model of ductal carcinoma calibrated to patient data to estimate biophysical parameters that are challenging to observe experimentally, such as time duration of apoptosis and cell calcification. Additionally, Rocha *et al*. [3] developed a hybrid three scale model consisting of a reaction-diffusion type continuum model of the tumor microenvironment (tissue scale), a lattice-free ABM of cell dynamics (cellular scale), and an inter- and intracellular signaling pathways model represented by a system of coupled nonlinear differential equations (sub-cellular scale). The model describes the major biological feature of tumor cell dynamics, such as the proliferative, hypoxic, and necrotic regions. For a comprehensive review of discrete and hybrid tumor growth models and their applications, the interested reader is referred to [33], and the references cited therein.

In spite of these advances in agent-based models, they still possess fundamental limitations that restrict their ability to accurately predict the evolution of a tumor given practical, experimental scenarios—a major goal of this developing field [34, 35]. In particular, the computational cost increases rapidly with the number of simulated agents since the evolution of the system relies upon the interactions of the individual cells with both each other and the surrounding milieu. This makes ABMs extraordinarily challenging for simulating large biological systems on practical time and length scales (see, e.g., [36, 37]). Another challenge arises when attempting to calibrate hybrid ABMs to experimental data as the models typically require measurements that span the micro- to macroscopic scales. Obtaining such measurements may be cost-prohibitive or not technically feasible. Furthermore, the intrinsic stochasticity of ABMs (due to the probabilistic decision criteria describing, for example, phenotypic transitions) adds further computational complications [38, 39]. Thus, standard parameter estimation techniques cannot adequately characterize the errors in ABM parameter calibrations [40], and new approaches are needed. Despite these barriers, there have been several previous efforts attempting to calibrate ABMs using experimental data. For example, Jiang *et al*. [5] showed that an ABM successfully captures the growth of mouse mammary tumor spheroids observed from in vitro measurements. Macklin *et al*. [26] calibrated a hybrid ABM using x-ray mammographic measurements of ductal carcinoma *in situ* and demonstrated the ability of the model to depict tumor heterogeneity. However, there are pronounced uncertainties in the parameters estimated from calibrating ABMs to experimental data, which translates into uncertainties in model predictions.

A common approach to reducing the high computational cost associated with hybrid ABMs is representing clusters of cells within the biological system by individual agents [31, 41–43], rather than simulating each cell with its own agent. This approach is known as “coarse-graining” in particle simulations of chemical or physical processes whereby several physical particles are lumped into a single simulation agent (or bead) to substantially reduce the degrees of freedom (see, e.g., [44]). Additionally, improving the predictive utility of hybrid ABMs in cancer requires the integration of data and models through a systematic model calibration and validation scheme that rigorously handles uncertainties in data and parameter calibration [25, 45, 46] as well as parameter inference methods that cope with the inherent stochasticity of the model [47, 48].

In this contribution, we overcome these challenges by developing a reduced-order ABM and implementing a parameter calibration method to address the stochasticity of the model. In particular, we establish a coarse-grained, two-scale ABM (cgABM) that reduces both the parameter space and computational cost, and can be adequately informed with a set of *in vitro*, time-resolved microscopy data of human breast carcinoma cells growing from a range of initial confluences and nutrient levels. By conducting a time-dependent variance-based global sensitivity analysis, we identify the relative importance of the parameters within the cgABM simulations. We then perform a Bayesian calibration to infer the cgABM parameters from the microscopy data while allowing for quantification of the uncertainties in model prediction due to model inadequacy and data uncertainty. The calibration is performed *via* a moment-based Bayesian inference that generalizes the likelihood function to account for the stochasticity of the cgABM in the inverse problem. Additionally, the Bayesian inferences are implemented using parallel codes with efficient use of high-performance computing resources that enable conducting the computationally expensive Bayesian calibration of the cgABM. Finally, the validity of our approach is assessed by predicting a set of measurements outside the calibration data.

## Materials and methods

### Experimental measurements

#### Cell lines and cell culture

BT474 human breast carcinoma cells were obtained from American Type Culture Collection (ATCC, Manassas, VA). The cells were grown in Dulbecco’s modified eagle medium (DMEM, Thermo Fisher Scientific Inc., Waltham, MA) supplemented with 10% fetal bovine serum (FBS, Sigma-Aldrich, St. Louis, MO) 1% L-glutamine (Thermo Fisher Scientific Inc.), and 1% Penicillin Streptomycin (Thermo Fisher Scientific Inc.) in 5% CO_2_ and air at 37°C.

D-(+)-glucose solution (Sigma-Aldrich) was added to glucose free DMEM to yield media with glucose concentrations of 2, 5, and 10 mM, which was not replenished during the experiments. For each glucose level there were three initial seeding densities, each with four replicates. Cells were seeded at a density of 3.5 × 10^4^, 5.0 × 10^4^ and 6.0 × 10^4^ cells/well on a 96-well tissue culture plate. We note that, in the present study, all references to “nutrient” in the model development presented above refer to glucose.

#### Time-resolved microscopy

Cells were incubated in the IncuCyte live-cell imaging system (Essen BioScience, USA). Multiple images were acquired with a 4× objective and automatically stitched together to obtain a whole well image for each well. The IncuCyte Cytotox Red reagent (Essen BioScience, USA), a highly sensitive cyanine nucleic acid dye, was added into the medium to quantify cell death. Once a cell’s plasma membrane begins to lose integrity, the cytotox red enters the cell and yields a 100–1000-fold increase in fluorescence upon binding to deoxyribonucleic acid (DNA). Phase-contrast images and fluorescent images (Red channel, excitation wavelength: 585 nm and emission wavelength: 635 nm) were acquired every 3 hours for 96 hours.

#### Image segmentation to quantify confluence over time

The BT-474 cells within the phase-contrast images at each time point were segmented in Matlab (The Mathworks, Inc., Natick, MA). The first step was to define a mask corresponding to the size of a well in a 96-well-plate from the IncuCyte Software (Essen BioScience, Ann Arbor, MI). The mask was applied such that the region of interest only included the area within each well. The masked region was converted to grayscale and with the Matlab function “colfilt”, we calculated the standard deviation of signal intensities within each 3-by-3 sliding block of the image to detect the edge of cell clusters. Following that, a Gaussian filter was applied to smooth the image which was then normalized to yield signal intensities between 0 and 1 (by dividing the value in each pixel by the highest signal intensity from each image). Next, with the Matlab function “imerode”, we shrank the clusters size and enlarged the holes to avoid losing open space within clusters. The image was then binarized *via* the function “im2bw”, while the functions “imclose” and “imopen” were used to fill holes in the interior of cell clusters and to smooth object contours, respectively. Finally, small objects were removed from the image *via* the function “bwareaopen”. After the image segmentation is completed, the confluence is calculated by summing the area of all the segmented objects (i.e., the cells) and dividing it by the area of the well.

#### Time-resolved microscopy data

**Fig 1:**
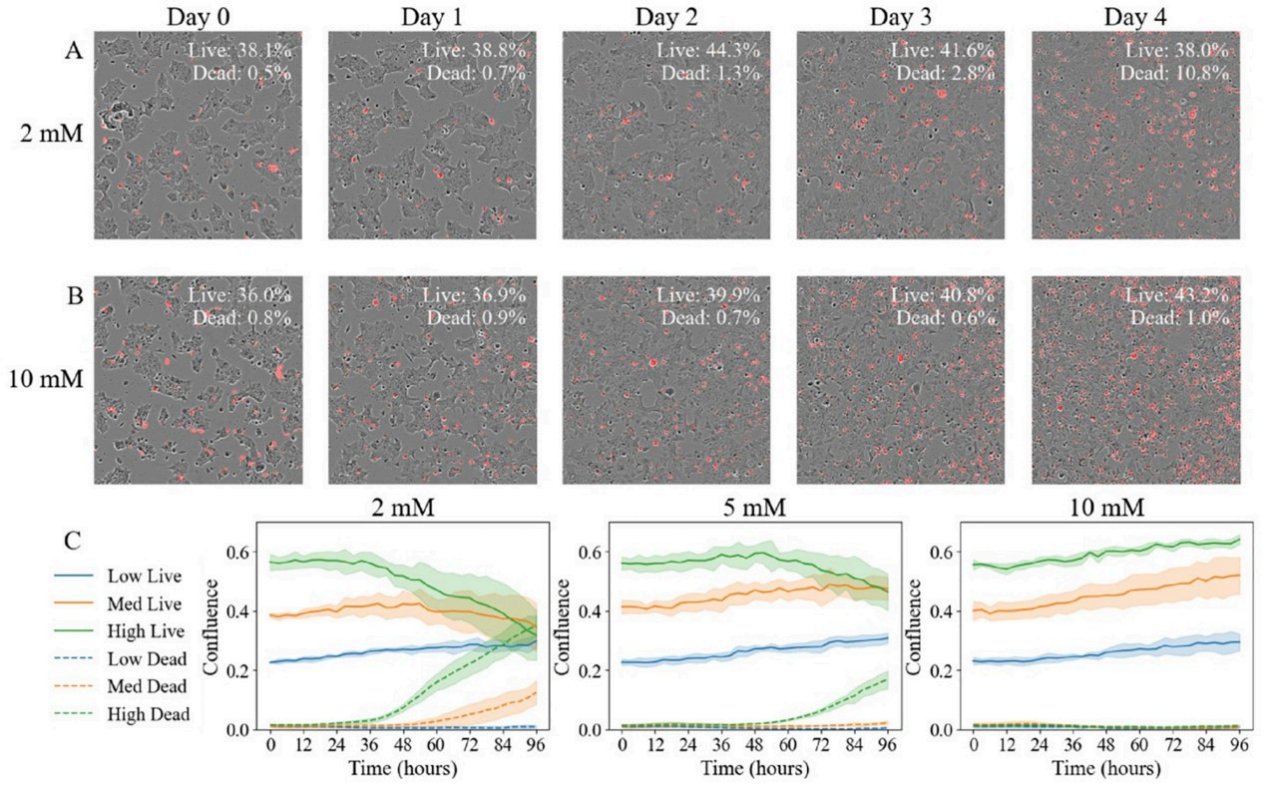
Time-resolved microscopy data. Row A presents example images from one well on days 0, 1, 2, 3, and 4 post seeding. While the whole-well image consists of 2240 × 2240 pixels, here we present a window of 400 × 400 pixels within the same area. These are merged images of phase contrast images and fluorescent images where dead cells were labeled in red by the Cytotox Red. Percent confluence of live and dead cells within the whole well are shown in the upper right corner of each panel. In this well, the cells were seeded at a density of 5.0 × 10^4^ cells/well, supplied with culture medium containing 2 mM glucose. With these initial conditions, the available nutrient allowed the tumor cells to increase in confluence until day 2, after which the environment cannot sustain growth, resulting in cell death. Row B presents example images from another well on days 0, 1, 2, 3, and 4 post seeding, where cells were seeded at the same density, supplied with culture medium containing 10 mM glucose. With these initial conditions, the available nutrient allowed the tumor cells to increase in confluence in 4 days. Each panel in row C presents confluences as a function of time (sampled every three hours) with the initial glucose level shown in the subtitle. In each panel, the average confluence of live cells with low, medium, and high seeding density are shown in blue, green, and orange solid lines, respectively, with the 95% confidence interval shown with shaded regions. The average confluence of dead cells with low, medium, and high seeding density are shown in blue, green, and orange dashed lines, respectively, with the 95% confidence interval shown with shaded regions.

Fig 1 displays a series of images showing tumor cell confluence over time in which the cells were seeded at a density of 5.0 × 10^4^ cells/well with either 2 mM (row A) or 10 mM glucose (row B). It is observed that as time progresses, the tumor cells in row A rapidly consume the glucose, yielding an environment that becomes somewhat unfavorable for continued expansion as manifested by the increase in dead cells (red) decays after day 2. Conversely, the cells seeded within 10 mM of glucose (row B) are able to continue to expand with minimal cell death. (See figure caption for more details).

### Hybrid two-scales agent-based model

The model we develop is a modification of a hybrid cell-tissue ABM we previously introduced [3] which links the tissue, cell, and sub-cell scales. Briefly, at the tissue level, the dispersion of nutrients and growth factors in the tumor microenvironment is modeled through reaction-diffusion equations. The ABM characterizes the cell level by describing normal and tumor cell dynamics, with cancer cells differentiated into proliferative, apoptotic, hypoxic, and necrotic states. Finally, the sub-cell scale integrates the epidermal growth factor receptor (EGFR) pathway as modeled by a system of coupled nonlinear differential equations. As our primary interest here is to calibrate this model to the time-resolved microscopy measurements of a growing tumor mass, we neglect the sub-cellular signaling pathway model, reduce the possible cell phenotypical states, and coarse-grain the discrete model so that each agent represents multiple cells with the same phenotypes. Additionally, to preserve the fidelity of the hybrid cgABM in depicting the observational measurements, the rule-based decisions of phenotypical transitions are enhanced compared to [3]. The details of the discrete cellular scale, the continuous tissue scale, and their coupling are summarized in the next subsections.

#### Discrete cellular scale model

The interactions between cells are captured by a discrete, lattice-free ABM in which the agents (i.e., a single tumor cell or a cluster of cells) are free to move throughout the domain unrestrained by a grid. As done in [3, 26, 28, 49], we represent the cell as a circle, and track the cell radius over time. The geometry of the *i*^*th*^ cell, at time *t* and position ***x***_*i*_, is defined by its radius *R*_*i*_, with an incompressible nucleus of radius $R_{i}^{N}$. We also define an action radius $R_{i}^{A}$ (with $R_{i}^{N}<R_{i}<R_{i}^{A}$) to specify short-range interactions capturing cell-cell adhesion and repulsion. In the cgABM, the cell radius, the cell nuclear radius, and the cell action radius are scaled by multiplying the single cell values by the square root of the number of cells per agent (i.e., the coarse-grained process). Thus, the area of the agent is proportional to the number of cells present. As the model does not track the shape of the cell, and assuming that cells that deform due to cell-cell interactions keep the same area, the tumor cell confluence is computed by summing the cell’s current area, disregarding overlap of the agents. The cell area changes over time during cell growth. When the cell divides, it splits into two cells, with each daughter cell having half of the area of the original cell. The radius of these cells changes over time until each daughter cell has the same area as the original cell. After the cell is fully grown, its area is not affected by interactions with surrounding cells. With these definitions, cell movement is determined by the following three mechanisms [3, 26]:

1. The cell-cell adhesive force, ***F***_*cca*_, and cell-cell repulsive force, ***F***_*ccr*_, between the *i*^*th*^ and *j*^*th*^ cells are defined as,
  $$\begin{array}{c} F_{cca}^{ij}=-c_{cca}\nabla \varphi (l^{ij};R_{i}^{A}+R_{j}^{A}), \end{array}$$ (1)
  $$\begin{array}{c} F_{ccr}^{ij}=-c_{ccr}\nabla \psi (l^{ij};R_{i}^{N}+R_{j}^{N},R_{i}+R_{j}), \end{array}$$ (2)
  where ***l***^*ij*^ = ***x***_*j*_ − ***x***_*i*_ is the distance between the center of the *i*^*th*^ and *j*^*th*^ cells, and *c*_*cca*_ and *c*_*ccr*_ are the cell-cell adhesion and repulsion scale parameters, respectively. The interaction potentials for adhesion, *φ*, and repulsion, *ψ*, are given by,
  $$\begin{array}{c} \nabla \varphi (l,R^{A})=\{\begin{array}{cc} {\left(\frac{\left|l\right|}{R^{A}}-1\right)}^{2}\frac{l}{\left|l\right|}, & 0\leq \left|l\right|\leq R^{A};\\ 0, & \text{otherwise}; \end{array} \end{array}$$ (3)
  $$\begin{array}{c} \nabla \psi (l,R^{N},R)=\{\begin{array}{cc} -(\frac{R^{N}\left|l\right|}{R^{2}}-\frac{2|l|}{R}+1)\frac{l}{\left|l\right|}, & 0\leq \left|l\right|\leq R^{N};\\ -(\frac{{\left|l\right|}^{2}}{R^{2}}-\frac{2|l|}{R}+1)\frac{l}{\left|l\right|}, & R^{N}\leq \left|l\right|\leq R;\\ 0, & \text{otherwise}. \end{array} \end{array}$$ (4)
  In Eq (3), the effects of the adhesion between the cells begins when their action radius overlaps, with the adhesion intensity increasing as the distance between the cells decreases. This phenomenon is balanced by the repulsion force, Eq (4), which begins to act when there is contact between two cells (i.e., the distance between the two cells is equal the sum of their radius). As we assume that cell nucleus is incompressible, the effects of the repulsion increases if the nucleus of the cells overlap.
2. The compression force, ***F***_*ct*_, and the resistance to the compression force, ***F***_*ccr*_, represent the effects of the boundary on the *i*^*th*^ cell as it grows and it is given by,
  $$\begin{array}{c} F_{ct}^{i}=-c_{ct}\nabla \varphi (l^{i};R_{i}^{A}), \end{array}$$ (5)
  $$\begin{array}{c} F_{rct}^{i}=-c_{rct}\nabla \psi (l^{i};R_{i}^{N},R_{i}), \end{array}$$ (6)
  where ***l***^*i*^ is the distance between the *i*^*th*^ cell and the domain boundary, and *c*_*ct*_ and *c*_*rct*_ are the cell-boundary adhesion and repulsion scale parameters, respectively. The simulation domain represents the whole experimental well. Therefore, we consider a non-permeable incompressible boundary, such that the tumor cells cannot leave the domain.
3. Due to the low speed of interstitial flow, the linear drag force of interstitial fluid flow, ***F***_*drag*_, is captured *via*,
  $$\begin{array}{c} F_{drag}^{i}=-\nu v_{i}, \end{array}$$ (7)
  where ***v***_*i*_ is the velocity of the *i*^*th*^ cell, and the constant *ν* characterizes the fluid viscosity.

The balance of forces acting on the *i*^*th*^ cell of mass *m*_*i*_ is obtained by Newton’s second law,

mivi˙=∑j=1j≠iN(t)(Fccaij+Fccrij)︷cell-cellinteraction+(Fdragi+Fcti+Frcti)︸cell-microenvironmentinteraction, (8)

where *N*(*t*) is the total number of cells. Disregarding the inertial effects and substituting the drag force from Eq (7) into Eq (8) results in the velocity of the *i*^*th*^ cell as

vi=1ν(∑j=1j≠iN(t)(Fccaij+Fccrij)+Fcti+Frcti). (9)

Thus, the position of the cell at time *t*_*k*+1_ is given as:

$$\begin{array}{c} x_{i}\left(t_{k+1}\right)=x_{i}\left(t_{k}\right)+v_{i}\Delta t, \end{array}$$ (10)

where Δ*t* = *t*_*k*+1_ − *t*_*k*_ indicates the time interval.

**Fig 2:**
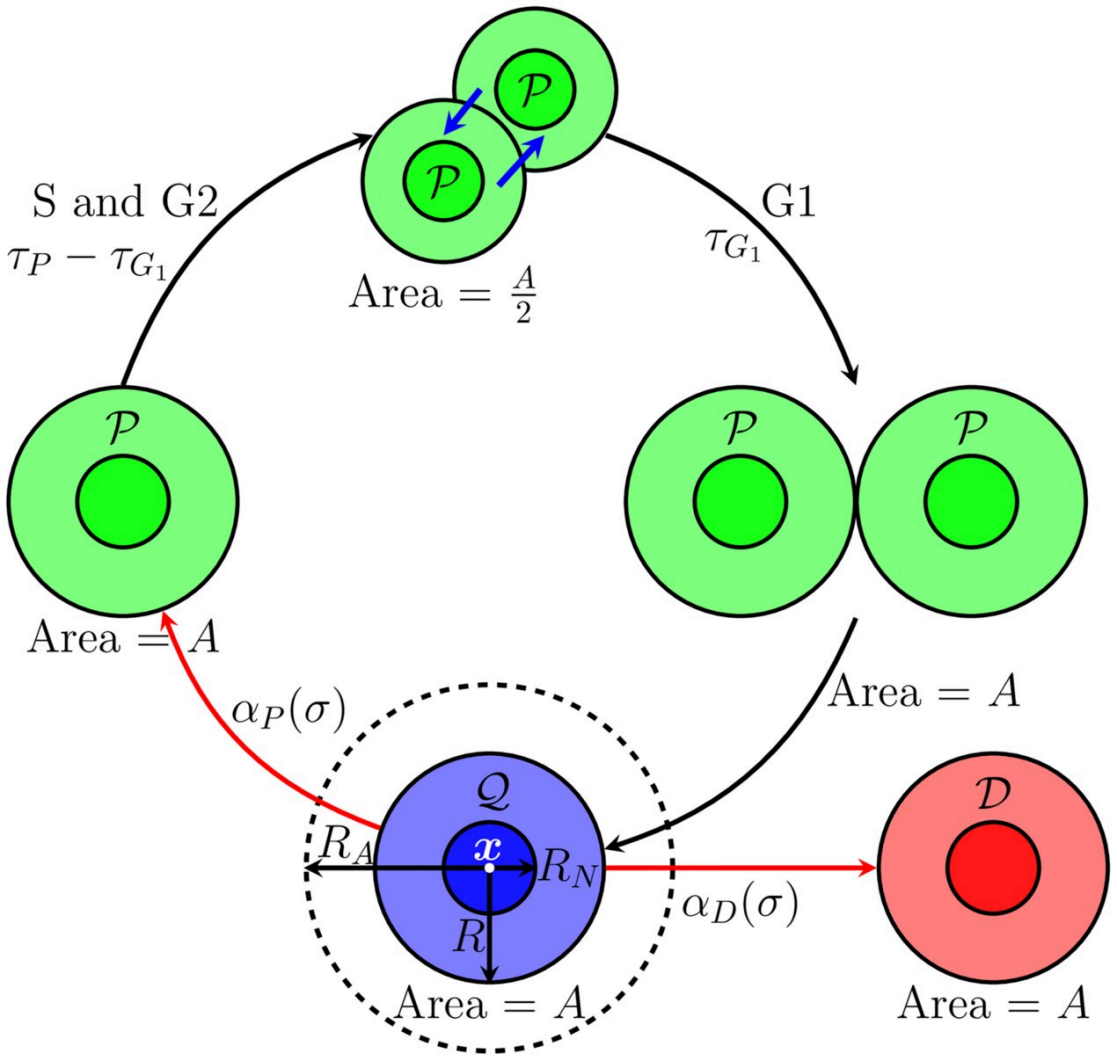
Schematic illustration of phenotypic transitions and geometric cell description. In the ABM, the geometry of the cell is approximated by a circle, where each agent stores the position of the cell (***x***), nucleus radius (*R*_*N*_), cell radius (*R*), and action radius *R*_*A*_. The cell radius is selected such that the area of the agent (*A*) is the same as the average cell area. The transitions between cell phenotypes can be deterministic (black arrows) or stochastic (red arrows). The transitions from quiescent cells (Q) are controlled by the intensity functions *α*_*P*_ and *α*_*D*_, which govern the transition to proliferative (P) and dead (D) cells, respectively. These transitions probabilities are proportional to the glucose concentration, *σ*. After a quiescent cell transition to the proliferative phenotype, the cells split into two cells with half of the original area after time interval *τ*_*P*_ − *τ*_*G*1_, where *τ*_*P*_ is the duration of the cell cycle, and *τ*_*G*1_ is the duration of the growth phase G1. This time interval includes the S and G2 phases of cell cycle. The adhesion and repulsion forces act on these new cells (blue arrows) to maintain the distance between them. The new cells enter the growth phase, reaching the same area as the original cell, and then return to the quiescent state.

In our model, the possible cell phenotypes are quiescent, Q, proliferative, P, and dying cells, D. Fig 2 provides a schematic illustration of rules for transitioning between these states. After a time *τ*_*P*_ − *τ*_*G*1_, (i.e., the differences between the duration of the cell cycle, *τ*_*P*_, and the duration of the growth phase, *τ*_*G*1_) the cell undergoes mitosis in which two daughter cells, each with half the area of the parent cell, are created in a deterministic process. The daughter cells grow until they reach the area of the parent cell, and enter the quiescent state after time, *τ*_*G*1_.

The transitions from the quiescent, Q, to the proliferative, P, and death, D, phenotypes are stochastic processes [3, 26, 49] and governed by the following probabilities:

$$\begin{array}{c} P(D|Q)=1-\text{exp}(-\alpha_{D}\left(\sigma \right)\Delta t), \end{array}$$ (11)

$$\begin{array}{c} P(P|Q)=1-\text{exp}(-\alpha_{P}\left(\sigma \right)\Delta t). \end{array}$$ (12)

**Fig 3:**
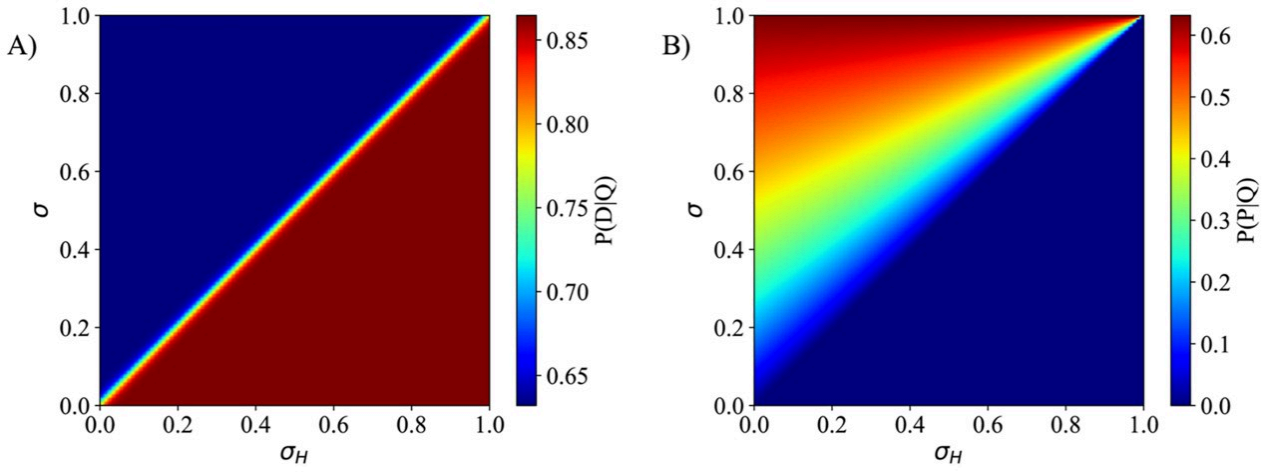
Transition probabilities of quiescent cells. Panels A) and B) present the dynamics of Eqs (13) and (14), respectively. We assume ${\bar{\alpha }}_{D}=1$, *γ*_*D*_ = 1, ${\bar{\alpha }}_{P}=1$, and *k* = 50. In panel A), the apoptosis probability has two constants values, below and above the glucose threshold, while *k* controls how smooth the transition is between these two constants. The apoptosis probability when *σ* > *σ*_*H*_ is proportional to ${\bar{\alpha }}_{D}$, and proportional to ${\bar{\alpha }}_{D}+\gamma_{D}$ when *σ* < *σ*_*H*_. In panel B), if the glucose concentration, *σ*, is below *σ*_*H*_, the proliferation probability is equal to zero. However, if *σ* > *σ*_*H*_, the probability increases as the glucose concentration increases.

In Eqs (11) and (12), the intensity factors *α*_*D*_ and *α*_*P*_ are functions of the nutrient concentration *σ*:

$$\begin{array}{c} \alpha_{D}\left(\sigma \right)={\bar{\alpha }}_{D}+\gamma_{D}\frac{1}{1+\text{exp}(-2k\left(\sigma_{H}-\sigma \right))}, \end{array}$$ (13)

$$\begin{array}{c} \alpha_{P}\left(\sigma \right)=\text{max}({\bar{\alpha }}_{P}\frac{\sigma -\sigma_{H}}{1-\sigma_{H}},0), \end{array}$$ (14)

where ${\bar{\alpha }}_{D}$ is the apoptosis rate, *γ*_*D*_ controls the increase in cell death due to the lack of nutrients, *σ*_*H*_ is the glucose threshold, and ${\bar{\alpha }}_{P}$ is the proliferation rate. The second term on the right-hand side in Eq (13) is a smooth approximation to the step function, such that a larger value of *k* leads to a sharper transition at *σ* = *σ*_*H*_. This term is incorporated to capture dynamics due to limited glucose availability, while its parameters can be well informed by the *in vitro* measurements of dead cell confluence. Eq (14) simulates the regulatory effect of the nutrient on cell proliferation up to the threshold *σ*_*H*_, below which the cell does not have enough nutrient to undergo mitosis. In this model, we assume that the oxygen levels are sufficient everywhere, so that glucose is the only limiting factor. In Fig 3, we present the effects of the glucose threshold in Eqs (13) and (14). While *σ*_*H*_ can be interpreted as the glucose threshold, the parameter actually represents the threshold beyond which there is an increase cell death due to an unfavorable environment. This value can change for different experimental scenarios as other factors might play a role in increasing cell death (e.g., cell crowding, death of surrounding cells, and other mechanical and chemical interactions). The phenotypic transition could be further extended to include, for example, the transition from proliferative to quiescent cells due to spatial constraints. However, we decided to simplify our model (and the subsequent required measurements) by considering the death of the cells due to unfavorable conditions (in this case, the lack of glucose) would partially capture this behavior.

#### Continuum tissue scale model

The nutrient dynamics (i.e., the temporal change in glucose concentration) is modeled at the tissue scale in which the nutrient is assumed diffuse in the microenvironment and taken up by living cells. Nutrient concentration, *σ*, is governed by a mass conservation condition based on reaction-diffusion equations. Let Ω be the domain with a smooth boundary *δ*Ω in which the development of living cells takes place due to the presence of nutrient, the governing equations at the macroscale is [3, 26, 49]:

$$\begin{array}{cc} \frac{\partial \sigma }{\partial t}=\nabla \cdot \left(D\nabla \sigma \right)-\Lambda \left(x,t\right)\sigma , & \;\text{in}\;\Omega , \end{array}$$ (15)

$$\begin{array}{cc} n\cdot \nabla \sigma =0, & \;\text{on}\;\partial \Omega , \end{array}$$ (16)

where *D* is the nutrient diffusion, ***n*** is a unit normal vector to *∂*Ω, and Λ(***x***, *t*) is a function describing the nutrient uptake rate. This function couples the discrete model with the continuum model (the cellular and tissue scales, respectively), and it is given as

$$\begin{array}{c} \Lambda \left(x,t\right)=\lambda \rho_{t}, \end{array}$$ (17)

where λ is the nutrient consumption rate by living cells (i.e., quiescent plus proliferative cells), and *ρ*_*t*_ is the volume fraction occupied by live cells. The nutrient uptake rate function averages the microscale (cellular) events.

#### Numerical solution of the hybrid ABM

The ABM is implemented in C++ using an object-oriented approach to model each cell in an object that stores the position (***x***, used in Eq (10)), velocity (***v***, computed in Eq (9)), and the forces acting on each cell (Eqs (1), (2), (5) and (6)). Additionally, the nuclear radius, cell radius, and the action radius are all saved to track each cell’s growth and compute the forces acting on the cells. The continuum reaction-diffusion model is solved using the C++ finite-element library libMesh [50]. The code itself, as a well as a description of how to use it, is provided at https://doi.org/10.6084/m9.figshare.15109209.v1. In summary, the solution steps of the hybrid ABM are:

1. Initialize the nutrient field uniformly according to the value used in the *in vitro* experimental condition (we normalized the initial condition by the highest value used experimentally).
2. Initialize the discrete model by seeding the tumor agents positions randomly in the domain such as the model confluences match the experimental live and dead cells tumor confluences. The fraction of proliferative cells among the live cells at the initial condition is between 0 and 1 (and determined by Eq (14)), and while the remaining live cells are quiescent.
3. Solve the reaction-diffusion equation (Eq (15)) and update the microenviroment conditions.
4. Update the phenotypic states based on the nutrient concentration.
5. Compute the balance of the forces acting on each cell (Eq (9)), and update cell positions (Eq (10)).
6. Return to step 3 and solve the model until the desired length of simulation is reached.

### Variance-based global sensitivity analysis

Global sensitivity analysis allows understanding and quantifying the impact of model parameters on the variation of the model outputs [51–53]. In time-dependent biological processes, such as those simulated by multiscale ABMs, sensitivity analysis determines the relative importance of each model parameter on the system responses during its evolution. In this regard, conducting sensitivity analysis aids in understanding the biological mechanisms that govern the system behavior. At the same time, it can guide the model calibration process by refining the estimations of the most effective parameters.

We employ a variance-based global sensitivity analysis method (also known as the Sobol’ Indices), in which the sensitivity of the model output to input parameters is computed by the quantity of (conditional) variance in the output caused by that specific input [54–56]. This method allows analyzing numerous model parameters simultaneously as well as being sufficiently general to handle complex multiscale problems. We now summarize the application of this method to our problem.

In a non-additive model, such as an ABM with *K* uncertain parameters (input factors) ${\left\{\theta_{k}\right\}}_{k=1}^{K}$, the model output **d**(***θ***) = **d**(*θ*_1_, *θ*_2_, ⋯, *θ*_*K*_) depends on the interactions among the parameters. The model output variance, V(d), can be decomposed by conditioning with respect to all inputs except *θ*_*k*_ [57, 58],

$$\begin{array}{c} V\left(d\right)=V_{\theta_{∼k}}(E_{\theta_{k}}\left(d|\theta_{∼k}\right))+E_{\theta_{∼k}}(V_{\theta_{k}}\left(d|\theta_{∼k}\right)), \end{array}$$ (18)

where *θ*_*k*_ is the *k*-th input factor, where ***θ***_∼*k*_ indicates the matrix of all factors except *θ*_*k*_, and $E_{\theta_{∼k}}\left(\cdot \right)$ and $V_{\theta_{∼k}}\left(\cdot \right)$ represents the mean and variance, respectively, taken over all possible values of ***θ***_∼*k*_ while *θ*_*k*_ is fixed. Eq (18) results in a sensitivity measure; the so-called *total effect index* [57, 58],

$$\begin{array}{c} S_{T_{k}}=1-\frac{V_{\theta_{∼k}}(E_{\theta_{k}}\left(d|\theta_{∼k}\right))}{V(d)}. \end{array}$$ (19)

In Eq (19), $V_{\theta_{∼k}}(E_{\theta_{k}}\left(d|\theta_{∼k}\right))$ denotes the expected reduction in variance if all values other than *θ*_*k*_ are fixed. Thus, the total effect, $S_{T_{k}}$, measures the contribution of the input *θ*_*k*_ to the model output variation. A “small” total effect index indicates that fixing *θ*_*k*_ at any value within the range of its uncertainty will not affect the model output significantly.

#### Monte-Carlo estimation of total effect sensitivity index

To compute the sensitivity index, we employ an efficient sampling method and an estimator proposed by Saltelli [52, 57, 59, 60]. Estimating $S_{T_{k}}$ using this method consists of constructing two *N* × *K* matrices, **A** and **B**, in which *N* random samples are drawn from a uniform distribution corresponding to the range of each parameters’ uncertainty. Additionally, *K* matrices $A_{B}^{\left(k\right)},\;k=1,2,\ldots ,K$, are defined where all columns are from **A** except the *k*^*th*^ column, which comes from **B**. The model outputs are then evaluated for each row of the matrices **A** and $A_{B}^{\left(k\right)}$ and the outputs are stored in the vectors ***Y***_***A***_ and $Y_{AB}^{\left(k\right)}$. The total-effect index for each parameter, ${\left\{S_{T_{k}}\right\}}_{k=1}^{K}$, can be approximated using the following estimator [60],

$$\begin{array}{c} S_{T_{k}}\approx \frac{1}{2N}\sum_{j=1}^{N}{\left({\left(Y_{A}\right)}_{j}-{\left(Y_{AB}^{\left(k\right)}\right)}_{j}\right)}^{2}. \end{array}$$ (20)

This algorithm reduces the computational cost of estimating multi-dimensional integrals to *N*(*K* + 1) model evaluations. For a time-dependent process, the above steps can be repeated for each time instance to represent the temporally-varying importance of each model parameter. We present the time evolution of $S_{T_{k}}$ for multiple ABM outputs (e.g., live and dead tumor cells with different nutrient and confluence initial conditions) in the Results section.

### Model calibration under uncertainty

Predictive modeling of biophysical systems requires characterizing the uncertainties in both the model parameters (due to simplifying assumptions made to develop the model) and the experimental data (due to noise and variability in measurements), as well as the uncertainty in the Quantity of Interest (QoI; i.e., the target of the prediction). Bayesian approaches to problems of statistical inference provide general frameworks for identifying the essential features of a predictive model, while also providing means to characterize uncertainty. The main feature of these approaches is that the model parameters, ***θ***, and the observational data, **D**, are random variables represented by probability density functions (PDFs), *π*(***θ***) and *π*(**D**), respectively. In this section, we first summarize a Bayesian calibration and validation process, and then discuss the form of the likelihood function for Bayesian inference of our stochastic ABM along with the numerical solution using a multi-level sampling algorithm.

#### Bayesian statistical inference

To represent the uncertainties in both the data and the model parameters, we make use of a statistical inference method in which the probability density functions (PDF) of the calibrated parameters are given by Bayes’ formula [61]:

$$\begin{array}{c} \pi_{\text{post}}\left(\theta |D\right)=\frac{\pi_{\text{like}}\left(D|\theta \right)\cdot \pi_{\text{prior}}\left(\theta \right)}{\pi_{\text{evid}}\left(D\right)}. \end{array}$$ (21)

In Eq (21), *π*_post_(***θ***|**D**) is the posterior PDF defining the Bayesian update of the prior information represented by *π*_prior_(***θ***), *π*_like_(**D**|***θ***) is the likelihood PDF, and *π*_evid_(**D**) is the evidence seen as a normalization factor (since ∫*π*_*post*_ = 1),

$$\begin{array}{c} \pi_{\text{evid}}\left(D\right)=\int \pi_{\text{like}}\left(D|\theta \right)\cdot \pi_{\text{prior}}\left(\theta \right)\;\text{d}\theta . \end{array}$$ (22)

One can use the principle of maximum entropy to construct the prior of the model parameters [62] based on their known features (e.g., bounds, mean, and variance). In the case that only the parameters’ bounds are available, then a uniform distribution is used as a prior ***θ***. Finally, to explain the posterior PDF, *π*_post_(***θ***|**D**), with a point estimate, one can use a Maximum A Posteriori (MAP) defined as,

$$\begin{array}{c} \theta^{\text{MAP}}=\underset{\theta }{\text{argmax}}\;\pi_{\text{post}}\left(\theta |D\right). \end{array}$$ (23)

#### Likelihood function for stochastic forward models

The form of the likelihood function reflects the way the discrepancy between the model output and the data are modeled. To account for uncertainties in computational models (i.e., model inadequacy) and measurement data (i.e., data noise), likelihood functions can be constructed by assigning a probability distribution, *p*_*ϵ*_, to the error representing the difference between the observational data, **D**, and the model output, **d**. The hybrid, agent-based model involves inherent randomness due to the stochastic processes defining the transition between the quiescent (Q), proliferative (P), and dead (D) states. Thus, the same set of parameter values, boundary conditions, and initial conditions will result in an ensemble of different outputs. The model output for the case of the stochastic model is represented by **d**(***θ***, *ω*), where *ω* ∈ Ω with Ω the set of possible outcomes. Under the additive noise assumption (see, e.g., [63–66]), the total error is described as ***ϵ*** = ***η*** + ***ξ*** = **D** − **d**(***θ***, *ω*), in which ***η*** and ***ξ*** indicate data noise and model inadequacy, respectively. Then the likelihood function is the probability density function describing the total error and is written as

$$\begin{array}{c} \pi_{\text{like}}\left(D|\theta \right)=p_{\epsilon }\left(D-d\left(\theta ,\omega \right)\right), \end{array}$$ (24)

where *p*_*ϵ*_ is a probability distribution. Here we assume that the error in the data and the model are Gaussian random variables with zero mean,

$$\begin{array}{c} \eta ∼N\left(0,\Gamma_{\text{data}}^{-1}\right),\;\xi ∼N\left(0,\Gamma_{\text{model}}^{-1}\right), \end{array}$$ (25)

where **Γ**_data_ and **Γ**_model_ are the covariance matrices.

To develop the likelihood for stochastic models, at each time step, *i* = 1, ⋯, *N*_*t*_, we denote a data point $D_{i}^{\left(j\right)}$ as a sample from a distribution, $D_{i}^{\left(j\right)}∼p\left(D\right)$ with *j* = 1, ⋯, *N*_*D*_. Similarly, to represent the randomness in the stochastic model, we consider $d_{i}\left(\theta ,\omega_{j}\right)=d_{i}^{\left(j\right)}$ as *j* = 1, ⋯, *N*_*r*_ independent identically distributed realizations of the model output at ${\left\{\omega_{j}\right\}}_{j=1}^{N_{r}}$ as samples from a distribution $d_{i}^{\left(j\right)}∼p\left(d|\theta \right)$. To represent the distance between the observational data **D** and the model output **d** in the likelihood function, we take the first moments of *p*(**D**) and *p*(**d**|***θ***). The sample estimates of the mean of the data and model are, respectively,

$$\begin{array}{ccc} \mu_{i}^{d}\left(\theta \right) & = & \frac{1}{N_{r}}\sum_{j=1}^{N_{r}}d_{i}^{\left(j\right)}\left(\theta \right), \end{array}$$ (26)

$$\begin{array}{ccc} \mu_{i}^{D} & = & \frac{1}{N_{D}}\sum_{j=1}^{N_{D}}D_{i}^{\left(j\right)}. \end{array}$$ (27)

We note that using sample-based estimates of the statistical moments with a finite number of model evaluations introduces statistical uncertainties in computing the means. While one can account for such uncertainties by approximating the variances of the moment estimators using methods of moment-based inference [67, 68], we consider sufficiently large *N*_*r*_ to minimize the statistical error as shown in the Results section. The assumptions in Eq (25) result in *p*_*ϵ*_ being a normal distribution $\epsilon ∼N(0,\Gamma_{\text{noise}}^{-1})$, where **Γ**_noise_ is the covariance matrix representing the data noise and model error. Assuming $\Gamma_{\text{data}}={\left(\sigma_{i}^{D}\right)}^{2}I$ and $\Gamma_{g}reys\text{model}={\left(\sigma_{i}^{d}\left(\theta \right)\right)}^{2}I$ in Eq (25), one can write

$$\begin{array}{c} \Gamma_{\text{noise}}={\left(\sigma_{i}\right)}^{2}I,\;{\left(\sigma_{i}\right)}^{2}={\left(\sigma_{i}^{D}\right)}^{2}+{\left(\sigma_{i}^{d}\left(\theta \right)\right)}^{2},\;i=1,2,\cdots ,N_{t}. \end{array}$$ (28)

Following the above considerations, the proposed likelihood function for the stochastic ABM can be written explicitly as

$$\begin{array}{c} \text{ln}\left(\pi_{\text{like}}\left(D|\theta \right)\right)=\sum_{i=1}^{N_{t}}(\frac{1}{2}\text{ln}\left(2\pi \right)-\text{ln}\left(\sigma_{i}\right)-\frac{1}{2}{\left(\frac{\mu_{i}^{d}\left(\theta \right)-\mu_{i}^{D}}{\sigma_{i}}\right)}^{2}). \end{array}$$ (29)

#### Sampling method for Bayesian inference

To conduct Bayesian inference, one needs to compute the posterior density *π*_post_(***θ***|**D**) as the solution of the statistical inverse problem. Typically, Markov Chain Monte Carlo (MCMC) sampling methods are employed to characterize the posterior distribution as they guarantee asymptotically exact recovery of the posterior distribution as the number of samples increases (see, e.g., [69, 70]). The solution of the Bayesian problem is computationally expensive as the posterior distribution may be a complex object requiring a large number of model evaluations. For the Bayesian calibration and validation of the ABM, we make use of a parallel, adaptive, multilevel MCMC algorithm [71]. In this work, we employ the adaptive multilevel MCMC implemented in the C++ library QUESO (Quantification of Uncertainty for Estimation, Simulation, and Optimization) [72] and refer the interested reader to [71] for details on the computational implementation of this method.

#### Error metric and model validation

To access the quality of the calibrated model in matching experimental data, we propose a metric using the cumulative probability distribution functions in $L^{1}\left(R\right)$. Let *ϕ*_*α*_ be the cell confluence with *α* = *L* or *D* (i.e., live or dead confluences, respectively). Also, Π^*d*^(*ϕ*_*α*_) and Π^*D*^(*ϕ*_*α*_) are the cumulative distribution functions for the model output and the measured data, respectively. The error metric is then given as

$$\begin{array}{c} M=\frac{\int_{-\infty }^{\infty }\left|\Pi^{d}\left(\phi_{\alpha }\right)-\Pi^{D}\left(\phi_{\alpha }\right)\right|\;\text{d}\phi_{\alpha }}{\Phi }, \end{array}$$ (30)

where Φ is the mean total confluence obtained from the experimental data. Eq (30) can be considered as the relative error; however, here we take into account the uncertainties of data and model prediction. Such error measure can be used to check whether a computational model is valid for predicting the quantities of interest. To this end, one must specify a tolerance (accepted) error for the model prediction *ε*_tol_. If the prediction error is below such tolerance, say,

$$\begin{array}{c} \frac{1}{N_{t}}\sum_{i=1}^{N_{t}}M_{t}\leq \varepsilon_{\text{tol}}, \end{array}$$ (31)

then the model is deemed to be valid and can be used for making predictions in scenarios well characterized by the calibration/validation data.

## Results

In all simulations, a circular domain of radius 3192 *μm* is used, which corresponds to the *in vitro* experimental domain. The computational domain, for the continuum model, is discretized by 2413 triangular elements with no flux permitted through the boundary. This boundary condition mimics the tumor and glucose being contained by the walls of the culture plate’s well. The timestep for the glucose and phenotypic transition is one hour, but, during this hour, the movement of the cells and the balance of the force is computed every minute (or until the cells reach equilibrium; whichever happens first).

### Developing a coarse-grained ABM

**Fig 4:**
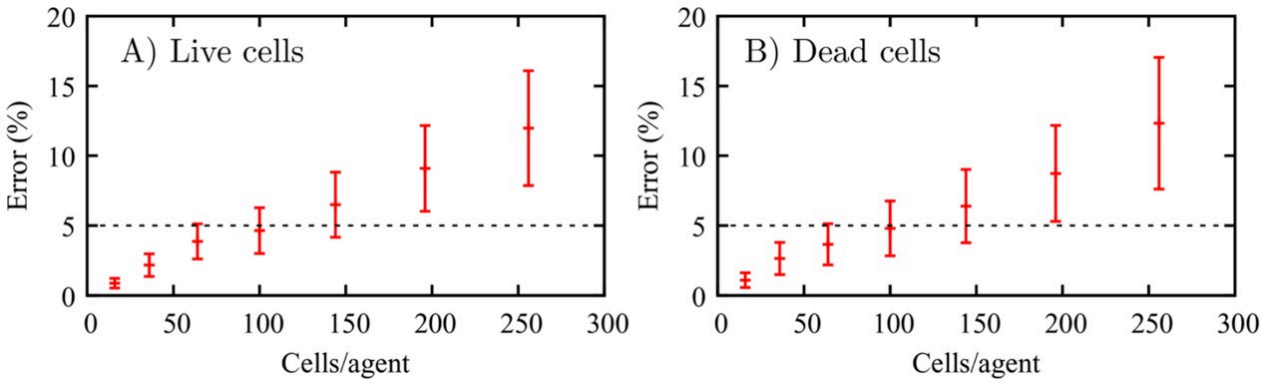
Effects of the coarse-graining on the ABM. Mean relative absolute error between the ABM and the cgABM, and its 95% credible interval (which is the Bayesian equivalent of the confidence interval), of live (A) and dead (B) cells. The simulations are executed for 16, 36, 64, 100, 144, 196, and 256 cells/agent. The horizontal dashed line indicates our 5% tolerance for the mean relative absolute error indicating that 100 cells/agent is the appropriate scenario for all subsequent analyses. The mean error and 95% credible interval between the coarse-grained model and the ABM, for the 100 cells/agent scenario, are 4.64 ± 1.64% and 4.80 ± 1.96% for live and dead cells, respectively.

The effects of the coarse-graining on the ABM simulations are restrained by the amount of relative error between the ABM and cgABM. Fig 4 shows the mean relative error and the 95% credible interval of live (Fig 4A) and dead (Fig 4B) cells confluence for different degrees of coarsening compared to the single-cell per agent. The simulations are conducted for 5 mM glucose concentration with the initial confluences of dead and live cells of 0.5 and 0.3, respectively. In the *in vitro* experiments, the average initial dead cell confluence (i.e., the fraction of the domain occupied by dead cells) is 0.029 ± 0.005, and the average percentage of dead cells at day = 0 is 6.5 ± 0.7% [73]. (We note that the cell viability in a healthy cell culture should be 80–95%; thus, the percentage of dead cells in our experiments is aligned with expectations [74].) However, we selected a high initial confluence of dead cells (0.3) so that we can observe the effects of the parameters on the confluence of dead cells from the beginning of the simulations. The values of the parameters used in these numerical experiments are defined in Table 1, and the tolerance for the desired mean relative absolute error is set at 5%. This threshold can be reduced based on the scenario being studied or the acceptable error in the model prediction. The highest degree of coarsening that satisfies this tolerance, both for live and dead cells, is the 100 cells/agent. It is readily apparent that increasing the degree of coarse-graining results in a higher error and, more importantly, higher variance in the cgABM simulations. Based on these results, all subsequent analyses are presented with a coarse-graining of 100 cells/agent.

**Table 1:** Model parameters.

Parameter	Physical meaning	Deterministic values coarse-graining studies	Distributions/Priors sensitivity analyses/calibration	How assigned
${\bar{\alpha }}_{P}$	Q→P transition rate	0.0493 *h*^−1^	$U\left(0,1\right)\;h^{-1}$	Calibrated
${\bar{\alpha }}_{D}$	Q→D transition rate	0.000408 *h*^−1^	$U\left(0,0.02\right)\;h^{-1}$	Calibrated
*D*	glucose diffusion coefficient	50 *μm*^2^/*h*	$U\left(0,100\right)\;\mu m^{2}/h$	Fixed
λ	glucose uptake rate	0.0483 *h*^−1^	$U\left(0,1\right)\;h^{-1}$	Calibrated
*γ*_*D*_	death rate increase	0.0245 *h*^−1^	$U\left(0,0.05\right)\;h^{-1}$	Calibrated
*k*	smooth transition constant	50	U(0,100)	Fixed
*σ*_*H*_	glucose threshold	0.0538	U(0,1)	Calibrated
*R*	cell radius	9.953 *μm*		Ref. [26]
*R*_*N*_	cell nuclear radius	5.295 *μm*		Ref. [26]
*R*_*A*_	action radius	1.214*R*		Ref. [26]
*c*_*ccr*_	cell-cell repulsion coefficient	10 *μm*/*min*		Ref. [26]
*c*_*cca*_	cell-cell adhesion coefficient	0.0489 *μm*/*min*		Ref. [26]
*τ*_*P*_	cell cycle time	18 *h*		Ref. [26]
*τ*_*G*1_	*G*_1_ cell cycle phase time	9 *h*		Ref. [26]
*τ*_*A*_	apoptosis time	8.6 *h*		Ref. [26]

Deterministic values of parameters used in the coarse-graining studies, parameter distributions for the sensitivity analyses, and priors for the Bayesian model calibration. The U(·,·) indicates the uniform probability distribution. The results of the sensitivity analysis indicate that *k* and *D* do not have a significant effect on live and dead cell confluences during the 96 hours simulated. Thus, these parameters are assumed to be constant during the model calibration.

**Fig 5:**
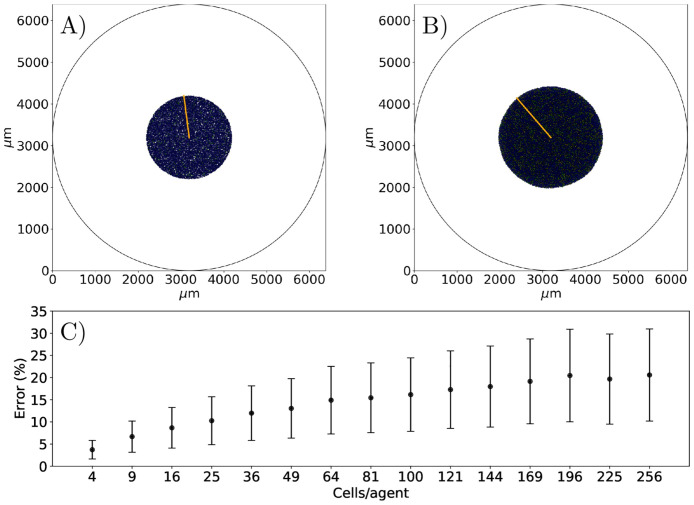
Error of the coarse-graining on the tumor radius. In panels A and B, snapshots of one realization of the ABM simulation at hours 0 and 96, respectively, conducted for 10 mM glucose concentration with an initial condition of 10000 cells. In panels A and B, the quiescent tumor cells are blue, dead cells are red, proliferative cells are green, the daughter cells are in yellow, and the orange line indicates the tumor radius. In panel C, we show the mean relative absolute error of 200 simulations with 15 different coarsening factor, and its standard deviation. For the 100 cells/agent, the error of the tumor radius is 16.15 ± 8.29%.

In the coarse-grained process, the cell radius (*R*), the cell nuclear radius (*R*_*N*_), and the cell action radius (*R*_*A*_) are scaled by multiplying the values in Table 1 by the square root of the number of cells per agent. In both ABM and cgABM, the movement of the agents is proportional to the forces acting on the cells (Eqs (1), (2), (5) and (6)), which are calculated using the distance between agents and their radii. In the coarse graining scenario, changing *R*, *R*_*N*_, and *R*_*A*_ is enough to keep the equilibrium distance between agents (i.e., the distance where the sum of the forces acting on the cell is zero) proportional to the distance of single-cell agents. Thus, there is no need to change any other values of the model parameters to keep the same movement dynamics in the cgABM as in the ABM. As an example, if we assume *c*_*cca*_ = 1 *μ*m/min, *c*_*ccr*_ = 0.5 *μ*m/min, *R*_*N*_ = 5 *μ*m, *R* = 10 *μ*m, and *R*_*A*_ = 12 *μ*m and insert these values into Eqs (3) and (4), the equilibrium distance between the agents (i.e., the distance where −(*c*_*cca*_ **∇** *φ* + *c*_*ccr*_ **∇** *ψ*) = 0) is set to 7.13 *μ*m from the center of the agent. However, if we scale the radius by 9 (i.e., *R*_*N*_ = 45 *μ*m, *R* = 90 *μ*m, and *R*_*A*_ = 108 *μ*m), the equilibrium distance is now at 64.17 *μ*m from the center of the new agent which is also 9× bigger than the previous example. Fig 5 shows the effects of the coarse-graining method on the spatial dynamics of the tumor and the mean relative absolute error of 15 coarse-graining scenarios compared to the single cell agent. This experiment indicates that, even though the error in the tumor confluence is below 5% for the 100 cells/agent, its error in the cluster’s radius is 16.15 ± 8.29%. As our quantity of interest is the tumor confluence (which disregards the spatial distribution of the cells), we continue the experiments with a coarse-graining of 100 cells/agent.

### Global sensitivity analysis

**Fig 6:**
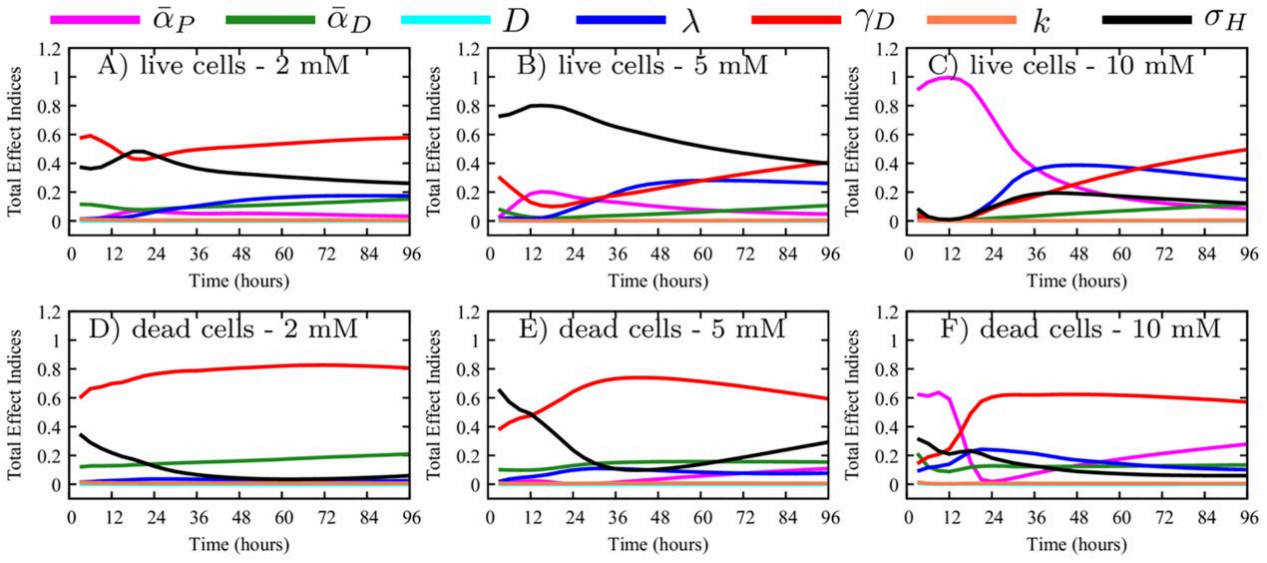
Medium confluence sensitivity analysis. Sensitivity analysis of proliferation rate (${\bar{\alpha }}_{P}$), death rate (${\bar{\alpha }}_{D}$), glucose diffusion (*D*), glucose uptake (λ), death rate increase due to lack of glucose (*γ*_*D*_), smooth transition constant (*k*), and glucose threshold (*σ*_*H*_) for live (top row) and dead (bottom row) cell phenotypes seeded with medium confluence. Panels A-F show the total effect index over time with Panels A, B, and C depicting live tumor cells, while Panels D, E, and F depict the dead tumor cells. The importance of the parameters is studied for three initial glucose concentrations: 2 mM (Panels A and D), 5 mM (Panels B and E), and 10 mM (Panels C and F). The glucose diffusion and the smooth transition constant have limited influence on the quantities of interest during the complete simulation (i.e., large changes in these parameters would yield small changes in tumor composition). Apart from these two parameters, the total effect index for every parameter is greater than 0.2 during the 96 hours simulated.

Variance-based global sensitivity analysis is conducted on the cgABM system to determine how each parameter contributes to the model outputs (i.e., the live and dead cell confluences). We perform the analysis for three different initial tumor confluences with the densities of 3.5 × 10^4^ (low), 5.0 × 10^4^ (medium), and 6.0 × 10^4^ (high) cells/well, as well as three initial glucose concentrations (2, 5, and 10 mM), and for seven parameters (*K* = 7) that we posit control the cell population (${\bar{\alpha }}_{P}$, ${\bar{\alpha }}_{D}$, *D*, λ, *γ*_*D*_, *k*, and *σ*_*H*_). The other model parameters (i.e., *R*, *R*_*N*_, *R*_*A*_, *c*_*ccr*_, *c*_*cca*_, *τ*_*P*_, *τ*_*G*1_, and *τ*_*A*_) are kept constant to the values reported in [26] (see Table 1). Due to the inherent stochasticity of the cgABM, the sensitivity analysis is performed on the sample estimate of the mean of the model output; i.e., substituting **d** in Eq (19) with *μ*^*d*^ from Eq (26), leading to a computational cost of *N*_*c*_ = *N*_*r*_*N*(*K* + 1). For estimating the total-effect index for each parameter in Eq (20), *N* = 1000 samples are drawn from the uniform distributions given in Table 1 with *N*_*r*_ = 16 model realizations per sample; therefore, the number of model evaluation required to obtain the total effect index (Eq (20)) is *N*_*c*_ = 128000 (for each initial condition). The convergence studies show that, when coarsening 100 cells/agent, *N*_*r*_ = 16 model realizations are able to represent the stochastic model accurately (i.e., with less than 0.2% mean relative absolute error when compared to the solution with *N*_*r*_ = 10000). Fig 6 shows the total effect index for each parameter over time, for live (Fig 6A–6C) and dead (Fig 6D–6F) cell confluences for the cases initially seeded with medium confluence (i.e., 5.0 × 10^4^ cells). The simulation starts with the fraction of proliferative cells (P) among the live cells at the initial condition being given by Eq (14), while the remaining live cells are quiescent (Q). The results of the sensitivity analyses in this figure indicate that the parameter *γ*_*D*_ (red line), which controls the rate of cell death due to lack of glucose, is the most critical parameter affecting the accumulation of dead cells during the simulation. Note that this parameter is also central to determining the time course of living cell confluence when the concentration of glucose is low. However, it is the nutrient threshold, *σ*_*H*_ (black line), that is the most important parameter for the live cell confluence when the initial nutrient concentration is 5 mM. The proliferation rate (${\bar{\alpha }}_{P}$, purple line) is the most influential parameter for the temporal development of live cells up to 36 hours, from 36 to 66 hours it is the glucose uptake rate (λ, blue line), while it is *γ*_*D*_ (red line) that is the most influential parameter at longer times (for the 10 mM initial glucose concentration). In Fig 6, the total effect indices of parameters *k* (orange line) and *D* (teal line) are consistently close to zero and never exceed 0.2. These results indicate that *k* and *D* do not have a significant effect on live and dead cell confluences during the 96 hours simulated. Thus, these parameters are assumed to be constant (i.e., the fixed values in Table 1) in the calibrations described in the next section. We note that these results are specific to the same scenarios as the *in vitro* experiments, and to the same quantities of interest (i.e., live and dead cells confluence), but are not general conclusions about the hybrid two-scale ABMs and the nutrient diffusion. In the scenarios tested here, the model could be further simplified by removing the spatial component of the nutrient equation. However, we decided to keep the term to have a general model which can incorporate spatial data in future studies. The sensitivity analysis for low and high initial confluences are shown in S1 and S2 Figs, respectively, and corroborate the conclusions obtained for the medium initial confluence. From Fig 6, S1 and S2 Figs, we conclude that the total effect indices of the parameters do not change for dead cells when the initial tumor confluence changes. However, for the live cells, these indices are affected by the initial tumor confluence. As the initial confluence increases, the importance of the nutrient uptake (λ) decreases, and the death rate due to lack of nutrients (*γ*_*D*_) increases. As an example, the total effect index of λ reaches 0.6 for the live cells with 10 mM glucose and low initial confluence, and it drops to 0.2 as the initial confluence increases to high (i.e., 6.0 × 10^4^ cells/well). The opposite behavior is observed for the *γ*_*D*_ total effect index, increasing from 0.2 to 0.6 as the initial confluence increases.

**Fig 7:**
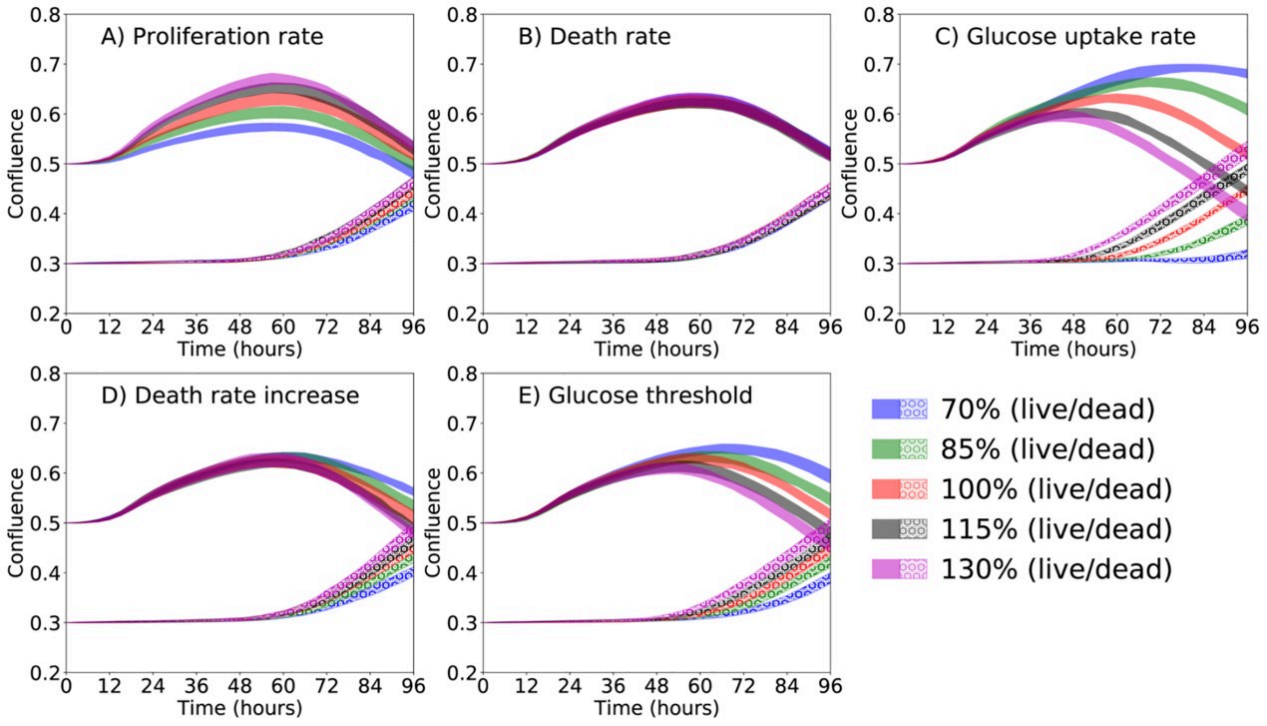
Parameter sweep. Effect of proliferation rate (${\bar{\alpha }}_{P}$; panel A), death rate (${\bar{\alpha }}_{D}$; panel B), glucose uptake (λ; panel C), death rate increase due to lack of glucose (*γ*_*D*_; panel D), and glucose threshold (*σ*_*H*_; panel E) for live and dead cell phenotypes. The live cells are seeded with 0.5 confluence, and the dead cells with 0.3 confluence. The base value of the parameters are the ones shown in Table 1 (from the deterministic values column). We changed one parameter at a time by 15% (reducing or increasing up to 30%). According to Panel A, the live and dead cell confluences are directly proportional to the proliferation rate. In Panels B, C, D, and E (i.e., for the death rate, glucose uptake, death rate increase due to lack of glucose, and glucose threshold, respectively), the live cell confluence is inversely proportional to the parameters being tested, while the dead cell confluence is directly proportional.

In Fig 7, a parameter sweep shows the effect of the parameters on the live and dead cell confluence. The base value of the parameters are the ones shown in Table 1 (from the deterministic values column). We changed one parameter at a time by 15% (reducing or increasing up to 30%). The live cells are seeded with 0.5 confluence, and the dead cells with 0.3 confluence. The tumor dynamics is measured during 96 hours. The results indicate that with an increased proliferation rate (${\bar{\alpha }}_{p}$; panel A), the confluence of both dead and live cells increase (Fig 7A). However, as we increase the death rate (${\bar{\alpha }}_{D}$; Fig 7B), glucose uptake rate (λ; Fig 7C), death rate increase (*γ*_*D*_; Fig 7D), and glucose threshold (*σ*_*H*_; Fig 7E) the live cell confluence decreases, while the dead cell confluence increases.

### Scenario-specific calibrations

Guided by the results from the sensitivity analysis, we proceed with the Bayesian calibration of the cgABM, using the *in vitro* experimental data. In particular, we will calibrate the proliferation rate, death rate, glucose uptake rate, death rate increase, and glucose threshold, $\theta =({\bar{\alpha }}_{P},{\bar{\alpha }}_{D},\lambda ,\gamma_{D},\sigma_{H})$, while holding the remaining parameters constant. Before calibrating the model with measurements, a parameter identifiability analysis of the cgABM (see S1 Appendix) was conducted to ensure the parameters can be inferred with high accuracy from perfect data. The priors for the parameters to be calibrated and the fixed values for the constant parameters are defined in Table 1. The uniform priors were selected such that the bounds on the distribution include the ranges in [3, 26, 28], and live and dead cell confluences were able to reach 1.0 (i.e., the whole well covered by live and/or dead cells) within 96 hours. As indicated above, the calibration data D consists of the time evolution of live and dead cell confluences for nine scenarios, comprised of three different initial glucose concentrations (2 mM, 5 mM, and 10 mM) and three different initial tumor confluences with the densities of 3.5 × 10^4^ (low), 5.0 × 10^4^ (medium), and 6.0 × 10^4^ (high) cells/well. As shown in Table 2, we utilize an abbreviation to refer to each scenario. For example, 2-M represent the scenario with 2 mM of initial glucose concentration and medium initial tumor cell confluence.

**Table 2:** Mean error and standard deviation.

Tumor initial condition	Glucose	Scenario name	Scenario-specific		Multi-scenario		Leave-one-out (Prediction)	
			Error live	Error dead	Error live	Error dead	Error live	Error dead
High	10 mM	10-H	1.79 ± 0.24	0.67 ± 0.08	11.10 ± 1.68	6.69 ± 1.50	6.22 ± 1.34	5.53 ± 1.60
High	5 mM	5-H	4.44 ± 0.68	1.73 ± 0.77	6.32 ± 1.12	2.46 ± 0.47	7.28 ± 1.89	4.74 ± 2.27
High	2 mM	2-H	6.43 ± 1.01	3.45 ± 1.15	6.00 ± 1.02	5.93 ± 1.70	13.97 ± 3.69	15.80 ± 5.59
Medium	10 mM	10-M	5.90 ± 0.43	1.71 ± 0.15	8.14 ± 0.72	6.45 ± 1.37	13.93 ± 2.77	7.32 ± 1.55
Medium	5 mM	5-M	4.96 ± 0.37	0.94 ± 0.15	5.09 ± 0.85	5.83 ± 1.40	15.20 ± 3.06	7.30 ± 1.67
Medium	2 mM	2-M	6.97 ± 1.24	2.11 ± 0.64	10.29 ± 1.83	5.09 ± 0.84	20.56 ± 2.85	12.86 ± 1.74
Low	10 mM	10-L	3.68 ± 0.52	1.84 ± 0.20	10.01 ± 1.26	6.44 ± 1.25	7.61 ± 2.01	5.78 ± 1.13
Low	5 mM	5-L	3.41 ± 0.33	1.98 ± 0.33	7.28 ± 1.75	7.27 ± 1.38	9.08 ± 2.00	4.99 ± 0.93
Low	2 mM	2-L	2.18 ± 0.31	2.10 ± 0.18	18.04 ± 3.47	9.37 ± 2.10	21.49 ± 4.27	9.56 ± 2.39
Mean average error			4.42 ± 1.72	1.84 ± 0.74	9.14 ± 3.71	6.17 ± 1.73	12.82 ± 5.36	8.21 ± 3.62

Computed error for the scenario-specific and multi-scenario calibration, and for the leave-one-out prediction. The nomenclature of the scenarios refers to the glucose concentration (2 mM, 5 mM, and 10 mM) followed by the initial tumor confluence (low—L, medium—M, and high—H). The last row indicates the mean average error over the nine experiments in each scenario.

In this section, we present the “scenario-specific calibration” process, in which the cgABM is calibrated for each of the nine measurement scenarios individually, resulting in nine sets of calibration posteriors of the model parameters (see S2 Appendix for the computational details of the Bayesian implementation). Such a calibration process is performed to ensure the developed cgABM can simulate the experimentally observed responses of cell evolutions and to investigate the effect of the initial glucose concentrations and tumor confluences on the estimated parameters. The cgABM calibrations using multiple experimental scenarios are presented in the following section.

**Fig 8:**
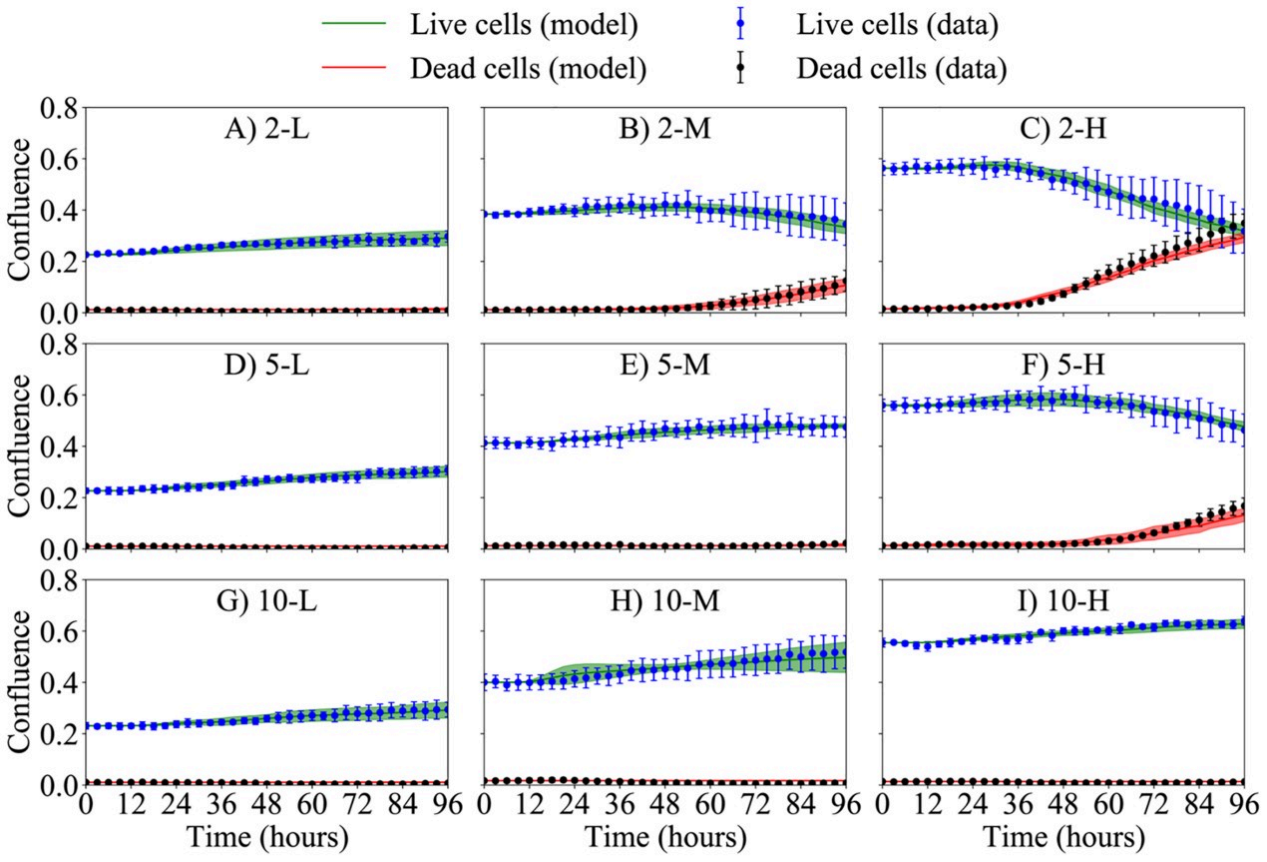
Calibration of scenarios with 2, 5, and 10 mM initial glucose concentration. Scenario-specific calibration of the cgABM to the time-resolved microscopy data for scenarios 2-L (panel A), 2-M (panel B), 2-H (panel C), 5-L (panel D), 5-M (panel E), 5-H (panel F), 10-L (panel G), 10-M (panel H), and 10-H (panel I). The data mean and 95% credible interval for the live and dead cells are shown in blue and black, respectively. The mean of the simulation is represented as a solid line, and the area is the 95% credible interval for the live (green) and dead (red) cells. The low initial confluence in scenario 2-L assures that the initial glucose concentration can sustain tumor growth during the 96 hours. The high initial confluence in scenario 5-H is the only one in the experiments with 5 mM initial glucose concentration for which the initial glucose concentration cannot sustain tumor growth during the 96 hours, leading to an increase in the dead cell confluence. The 10 mM glucose condition is sufficient to sustain tumor growth during the 96 hours for all seeding density tested.

Fig 8 compares the calibrated cgABM results with the *in vitro* data for 2 mM, 5 mM, and 10 mM initial glucose concentrations. The error bars show the data uncertainty due to the four replicates of the experimental measurements. The prediction results in this figure are obtained from 200 parameter samples drawn from the calibration posteriors and computing the mean of the cgABM simulations (solid line) and 95% credible interval (the green and red areas for live and dead cells respectively). Thus, the uncertainty in the model predictions is due to the stochasticity of the cgABM as well as the parameter uncertainty. Table 2 presents the error between the model outputs and the experimental measurements according to Eq (30), and includes means and the 95% credible interval. Due to insufficient glucose for the higher tumor cell numbers, the scenarios 2-M, 2-H, and 5-H exhibit an increase of dead cells over time. As expected, the confluence of dead cells does not significantly increase with time in the scenarios with higher glucose concentration and lower initial tumor cell confluence. The scenario-specific calibration errors shown in Table 2 indicate that the model is able to capture the evolution of live and dead cells with an average error below 7%. Moreover, the model can successfully predict the increase of dead cells when glucose is consumed, as in scenarios 2-M, 2-H, and 5-H. The maximum discrepancy between data and model occurs in the 2-H scenario and the smallest mean error for the case of 5-H.

**Fig 9:**
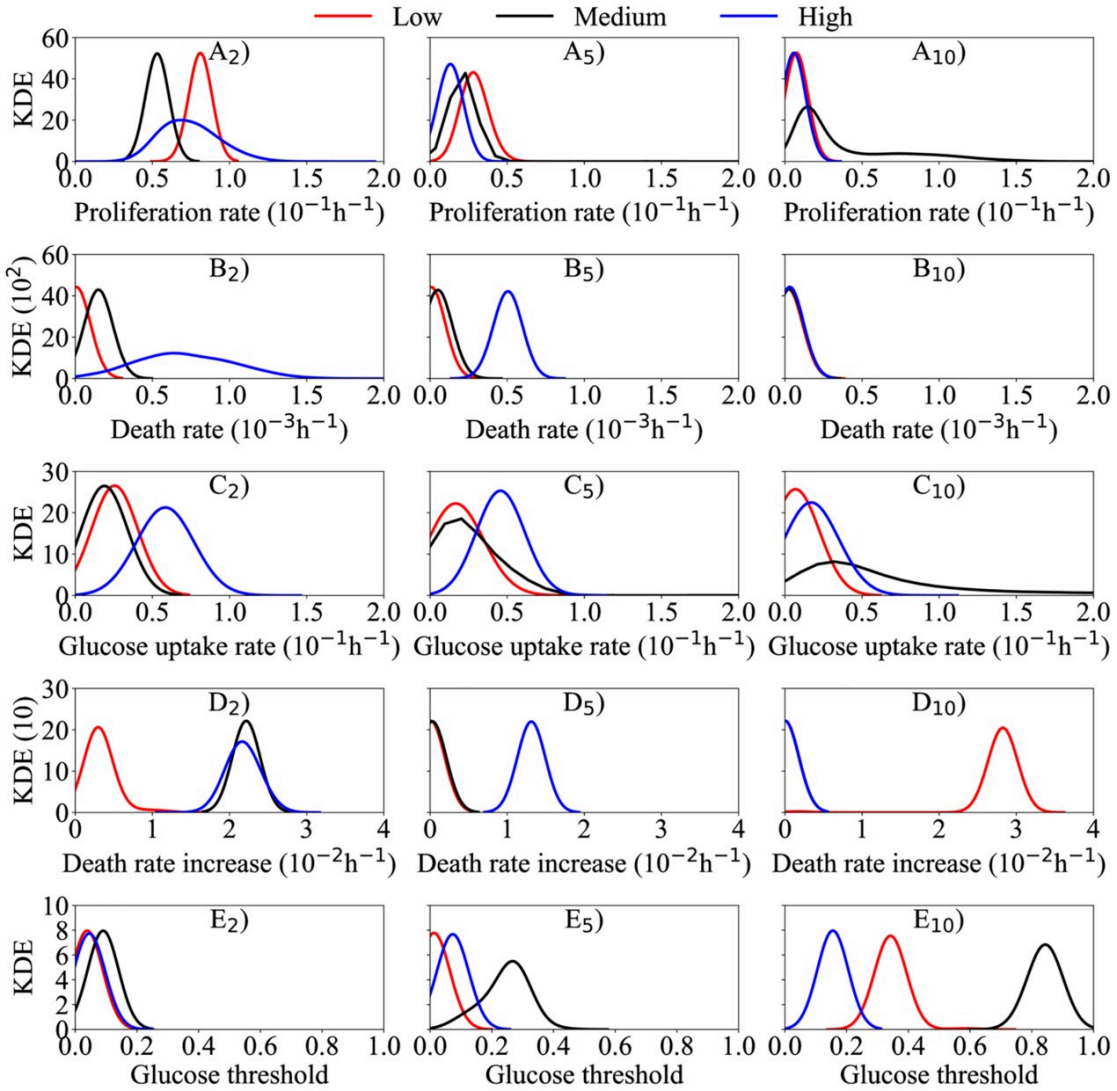
Posterior kernel density estimation (KDE) of scenarios with 2, 5, and 10 mM initial glucose concentration. The KDE obtained during the scenario-specific calibration of the 2, 5, and 10 mM initial glucose concentration (subscripts 2, 5, and 10, respectively) and low (red), medium (black), and high (blue) tumor initial condition for the following parameters: ${\bar{\alpha }}_{P}$ (proliferation rate—panel A), ${\bar{\alpha }}_{D}$ (death rate—panel B), λ (glucose uptake rate—panel C), *γ*_*D*_ (death rate increase—panel D), and *σ*_*H*_ (glucose threshold—panel E). For the 2 mM initial glucose concentration, the parameter *γ*_*D*_ does not influence the dynamics of the model and cannot be calibrated in the 2-L scenario as the glucose concentration is enough to sustain tumor growth and avoid the increase of the confluence of dead cells. For the 5 mM initial glucose concentration, the parameters ${\bar{\alpha }}_{D}$ and *γ*_*D*_ are different than zero just in the 5-H scenario. In this initial condition, the high initial confluence demands more glucose than it is available, leading to an increase of the dead cells confluence. This dynamic does not happen in scenarios 5-L and 5-M, where the initial glucose concentration is enough to sustain tumor growth. The 10 mM initial glucose concentration is enough to sustain tumor growth to all initial tumor confluences, such as that no increase in the confluence of dead cells is noticed. Due to the lack of the dead cells, and the fact that the glucose uptake rate is low, the glucose concentration never drops below *σ*_*H*_, and the parameter *γ*_*D*_ does not play a role on the tumor dynamics.

Fig 9 presents the kernel density estimation of the Bayesian calibration posteriors for all nine scenarios. Each panel summarizes scenarios with the same initial glucose concentration with low, medium, and high initial tumor confluences. For quantitative comparisons of the calibrated parameters in each scenario, we estimate the Maximum A Posteriori (MAP) points (see Eq (23)) for the MCMC samples of the posterior. We next summarize observations from the posteriors of the parameters in the scenario-specific calibration process: the proliferation rate (${\bar{\alpha }}_{P}$—Fig 9A), death rate (${\bar{\alpha }}_{D}$—Fig 9B), glucose uptake rate (λ—Fig 9C), death rate increase (*γ*_*D*_—Fig 9D), and glucose threshold (*σ*_*H*_—Fig 9E).

As the initial glucose concentrations increase from 2 mM to 10 mM, the MAP of the proliferation rate (${\bar{\alpha }}_{P}$) decreases. In particular, from 2 mM to 5 mM the average MAP of ${\bar{\alpha }}_{P}$ decreases 68%, and from 5 mM to 10 mM it decreases 58%. This trend is consistent with the function describing the proliferation rate, which is a function of ${\bar{\alpha }}_{P}$, *σ*_*H*_ (Eq (14)) and the current glucose concentration. The average MAP and standard deviation from 2 mM, 5 mM, and 10 mM are 6.74 × 10^−2^ ± 1.13 × 10^−2^ h^-1^, 2.16 × 10^−2^ ± 0.61 × 10^−2^ h^-1^, and 0.91 × 10^−2^ ± 0.35 × 10^−2^ h^-1^, respectively. Note how the variance of ${\bar{\alpha }}_{P}$ is smaller for the scenario with an initial glucose concentration of 10 mM (Fig 9). Once sufficient glucose is available for all proliferating cells (i.e., the 10 mM case), the proliferation rate is relatively constant with changes in initial tumor cell confluence. Additionally, the calibrated proliferation rate in Fig 9 varies non-monotonically in the 2-H, 2-M, and 2-L scenarios (Fig 9A_2_). In the 2-H scenario, this behavior is due to competition between cell proliferation (${\bar{\alpha }}_{P}$) and cell death (${\bar{\alpha }}_{D}$). The higher variance observed in the posterior of ${\bar{\alpha }}_{P}$ and ${\bar{\alpha }}_{D}$ for the 2-H scenario is a result of the higher number of dead cells (twice the number of dead cells than the scenario with the second highest value) observed in Fig 8C.

As the confluence of the dead cells is evident only in three scenarios, 2-M, 2-H, and 5-H (Fig 8), the average MAP and standard deviation of the death rate, ${\bar{\alpha }}_{D}$, is 2.32 × 10^−5^ ± 1.47 × 10^−5^ h^-1^ when disregarding these scenarios. For scenarios 2-M, 2-H, and 5-H, the MAP point is 1.53 × 10^−4^ h^-1^, 6.52 × 10^−4^ h^-1^, and 5.07 × 10^−4^ h^-1^, respectively. The higher MAP values of ${\bar{\alpha }}_{D}$ are due to the limited availability of glucose for these three cases, in which a greater number of proliferative cells cause a faster consumption of the glucose and subsequent increase of dead cells due to glucose depletion. Additionally, for the scenarios with 10 mM of initial glucose, ${\bar{\alpha }}_{D}$ remains the same with respect to the initial tumor confluences shown in Fig 9. The constant values of ${\bar{\alpha }}_{D}$ are because the tumor cells are in favorable glucose environments in scenarios 10-L, 10-M, and 10-H, and no increase in dead cells is observed (see Fig 8). Furthermore, similar to the ${\bar{\alpha }}_{P}$, the 2-H scenario represents the largest variance in the ${\bar{\alpha }}_{D}$ posterior, due to the distinct increase of dead cells in the observational data of Fig 8.

The glucose uptake rate (λ), death rate increase (*γ*_*D*_), and glucose threshold (*σ*_*H*_) parameter are strongly correlated due to their indirect relations to the glucose field. The parameter λ in the continuum model (see Eq (17)) controls how fast the glucose concentration decreases. The parameter *γ*_*D*_ controls the increase in cell death due to the lack of nutrients. Finally, *σ*_*H*_ is the glucose threshold below which cells do not have enough glucose to undergo mitosis (see Eq (14)). Due to these relations, a change in one parameter affects the value of the others. For example, if the cells have a higher uptake rate, that would lead to a lower glucose concentration and therefore lead to lower values of death rate increase and glucose threshold required to keep a similar number of dead cells. In all scenarios, the glucose uptake rate λ is proportional to the initial tumor cell confluence, in which a higher number of tumor cells leads to an increase in glucose consumption rate. The average MAP value of λ decreases as the glucose concentration increases, with a 20% decrease in the average MAP from 2 mM to 5 mM, and 32% from 5 mM to 10 mM. Only in the scenarios 2-M, 2-H, and 5-H, do the glucose levels drop below the threshold *σ*_*H*_, leading to activation of the cell death mechanism due to the lack of nutrients (the second term on the right-hand side of Eq (13)). This observation indicates that *γ*_*D*_ does not affect the evolution of the tumor cells in the other six scenarios (i.e., 2-L, 5-L, 5-M, 10-L, 10-M, and 10-H).

**Fig 10:**
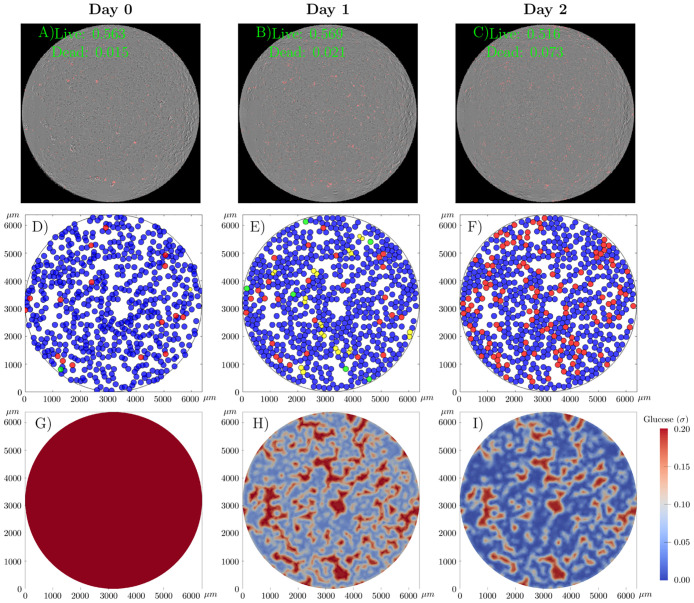
cgABM simulation for the scenario 2-H. Snapshots of one realization of the cgABM simulation for the scenario with high initial tumor cell confluence and 2 mM initial glucose (2-H). The whole-well images from three days of data (panels A, B, and C) show the merged images of phase contrast images and fluorescent images where dead cells were labeled in red by the Cytotox Red. The data present here is one of the four replicates of this scenario. In panels A, B, and C, the confluence of live and dead cells is presented. Spatial-temporal simulations of tumor cells (panels D, E, and F) and glucose concentration (G, H, and I) over three days. Panels A, D, and G display the initial conditions, while panels B, E, and H present the data and model at day 1, and panels C, F, and I display the data and model at day 2. The numerical simulation was performed using the MAP points of the calibrated parameters from the 2-H scenario (${\bar{\alpha }}_{P}^{\text{MAP}}=5.50\times 10^{-3}\;h^{-1}$, ${\bar{\alpha }}_{D}^{\text{MAP}}=6.52\times 10^{-4}\;h^{-1}$, λ^MAP^ = 6.37 × 10^−3^ *h*^−1^, $\gamma_{D}^{\text{MAP}}=2.18\times 10^{-3}\;h^{-1}$, and $\sigma_{H}^{\text{MAP}}=3.63\times 10^{-3}$). In panels D, E, and F, the quiescent tumor cells are blue, dead cells are red, proliferative cells are green, and the daughter cells are in yellow. As glucose is consumed by the tumor cells, its concentration drops below the threshold needed for proliferation, leading to an increase in dead cells by day 2, which is consistent with the data present in panel C. The results presented here are generated from one simulation using the MAP points. Due to the stochastic nature of the model, each run of the model produces a different result. The average error of the live and dead cells are 6.43 ± 1.01 and 3.45 ± 1.15, respectively.

Fig 10 shows snapshots of one realization of the cgABM simulation for the 2-H scenario (i.e., high initial tumor confluence and 2 mM of initial glucose concentration). For this simulation, we use the MAP estimates of the parameter posteriors from scenario 2-H (Fig 9), which are ${\bar{\alpha }}_{P}^{\text{MAP}}=5.50\times 10^{-3}\;h^{-1}$, ${\bar{\alpha }}_{D}^{\text{MAP}}=6.52\times 10^{-4}\;h^{-1}$, λ^MAP^ = 6.37 × 10^−3^ *h*^−1^, $\gamma_{D}^{\text{MAP}}=2.18\times 10^{-3}\;h^{-1}$, and $\sigma_{H}^{\text{MAP}}=3.63\times 10^{-3}$. The cgABM simulation in Fig 10 begins with confluences of 0.56 and 0.02 for the quiescent and dead cells, respectively, and a uniform glucose distribution of 2 mM. With the available glucose, the tumor cells proliferate, leading to the presence of growing daughter cells on day 1. The combination of an initial low glucose concentration, and high tumor confluence, yield a rapid decrease of glucose below the level required to maintain cell viability. By day 2, dying cells appear in the cgABM simulation, in agreement with the increase in dying cells observed in the experimental data (Fig 8).

**Fig 11:**
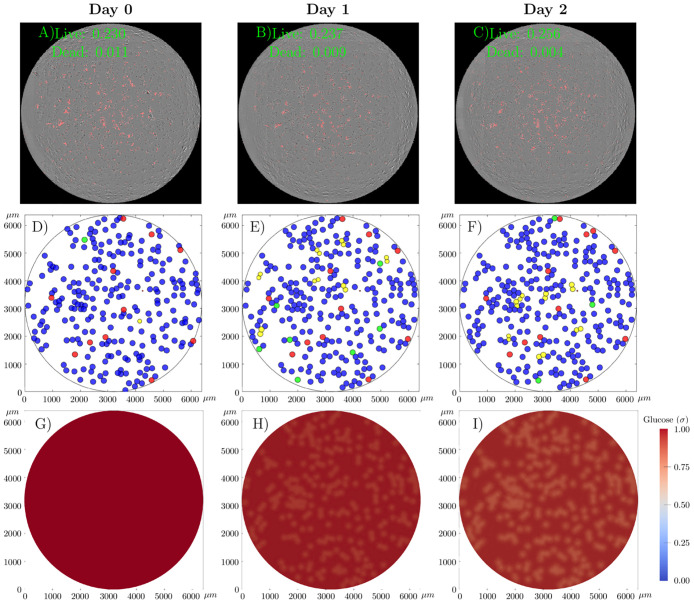
cgABM simulation for the scenario 10-L. Snapshots of one realization of the cgABM simulation for the scenario with low initial tumor cell confluence and 10 mM initial glucose (10-L). The whole-well images from three days of data (panels A, B, and C) show the merged images of phase contrast images and fluorescent images where dead cells were labeled in red by the Cytotox Red. The data present here is one of the four replicates of this scenario. In panels A, B, and C, the confluence of live and dead cells is presented. Spatial-temporal simulations of tumor cells (panels D, E, and F) and glucose concentration (G, H, and I) over three days. Panels A, D, and G display the initial conditions, while panels B, E, and H present the data and model at day 1, and panels C, F, and I display the data and model at day 2. The numerical simulation was performed using the MAP points of the calibrated parameters from the 10-L scenario (${\bar{\alpha }}_{P}^{\text{MAP}}=7.31\times 10^{-3}\;h^{-1}$, ${\bar{\alpha }}_{D}^{\text{MAP}}=1.73\times 10^{-4}\;h^{-1}$, λ^MAP^ = 3.44 × 10^−3^ *h*^−1^, $\gamma_{D}^{\text{MAP}}=8.45\times 10^{-3}\;h^{-1}$, and $\sigma_{H}^{\text{MAP}}=1.94\times 10^{-1}$). In panels D, E, and F, the quiescent tumor cells are blue, dead cells are red, proliferative cells are green, and the daughter cells are in yellow. The low tumor cell confluence does not reduce the glucose to values below *σ*_*H*_. Thus, the number of dead cells does not increase due to the lack of glucose. The average error of the live and dead cells are 3.68 ± 0.52 and 1.84 ± 0.20, respectively.

Fig 11 shows snapshots of one realization of the cgABM simulation for the 10-L scenario (i.e., low initial tumor confluence and 10 mM of initial glucose concentration). For this simulation, we use the MAP estimates of the parameter posteriors from scenario 10-L (Fig 9), which are ${\bar{\alpha }}_{P}^{\text{MAP}}=7.31\times 10^{-3}\;h^{-1}$, ${\bar{\alpha }}_{D}^{\text{MAP}}=1.73\times 10^{-4}\;h^{-1}$, λ^MAP^ = 3.44 × 10^−3^ *h*^−1^, $\gamma_{D}^{\text{MAP}}=8.45\times 10^{-3}\;h^{-1}$, and $\sigma_{H}^{\text{MAP}}=1.94\times 10^{-1}$. The cgABM simulation in Fig 11 begins with confluences of 0.23 and 0.01 for the quiescent and dead cells, respectively, and a uniform glucose distribution of 10 mM. The final confluence for the quiescent and dead cells, for this realization of this stochastic simulation, is 0.28 and 0.01, respectively. With this low tumor cell confluence, the glucose never drops below *σ*_*H*_, such that there is no increase in cell death due to the lack of glucose.

**Fig 12:**
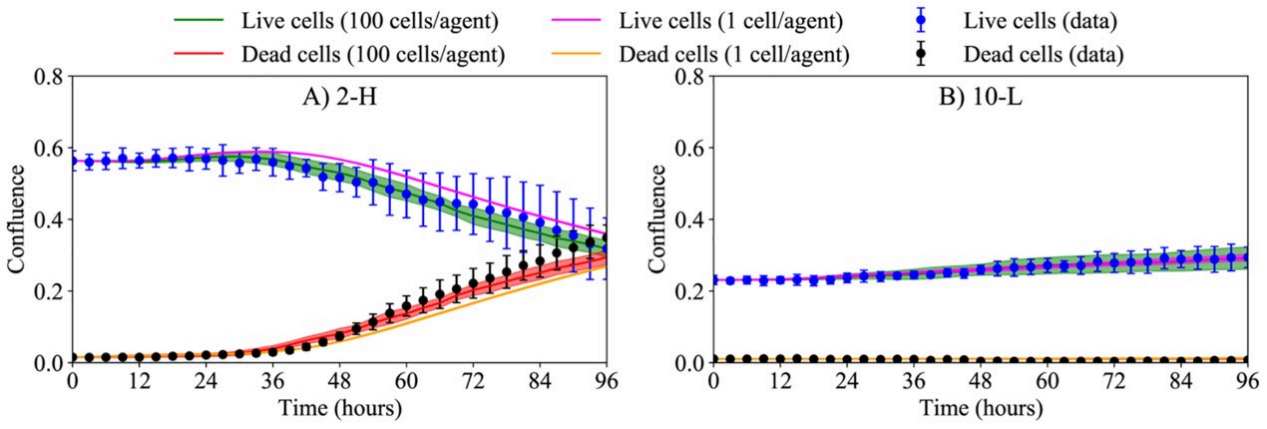
Comparison between the single-agent ABM using the parameter values from the cgABM, and the experimental data of scenarios 2-H and 10-L. Scenario-specific calibration of the cgABM to the time-resolved microscopy data for the 2 mM initial glucose concentration and high initial tumor cell confluence (panel A), and for the 10 mM initial glucose concentration and low initial tumor cell confluence (panel B). The data mean and 95% credible interval for the live and dead cells are shown in blue and black, respectively. The mean of the cgABM simulation is represented as a solid line, and the area is the 95% credible interval for the live (green) and dead (red) cells. The mean of the ABM simulation is represented as a solid line, and the area is the 95% credible interval for the live (magenta) and dead (orange) cells. The average error and standard deviation for live and dead cells, in the cgABM, in scenario 2-H are 6.43 ± 1.01% and 3.45 ± 1.15%, respectively. While that for the ABM using the parameter values of the cgABM are 8.19 ± 1.19% and 4.80 ± 1.50%, respectively. The average error and standard deviation for live and dead cells, in the cgABM, in scenario 10-L are 3.68 ± 0.52% and 1.84 ± 0.20%, respectively. While that for the ABM using the parameter values of the cgABM are 5.12 ± 0.63% and 1.96 ± 0.23%, respectively.

Fig 12 compares the calibrated cgABM, and the ABM using the calibrated parameter values from the cgABM, with the experimental data for two representative cases of the experimental data: 1) the 2 mM initial glucose concentration and high initial tumor cell confluence (Fig 12A), and 2) the 10 mM initial glucose concentration and low initial tumor cell confluence (Fig 12B). The errors from the cgABM and the ABM have the same order of magnitude. The average error and standard deviation for live and dead cells, in the cgABM, in scenario 2-H are 6.43 ± 1.01% and 3.45 ± 1.15%, respectively. While that for the ABM using the parameter values of the cgABM are 8.19 ± 1.19% and 4.80 ± 1.50%, respectively. The average error and standard deviation for live and dead cells, in the cgABM, in scenario 10-L are 3.68 ± 0.52% and 1.84 ± 0.20%, respectively. While that for the ABM using the parameter values of the cgABM are 5.12 ± 0.63% and 1.96 ± 0.23%, respectively. Importantly, the main difference between the cgABM and the ABM is the computational cost. While a single run of the cgABM for the 2-H and 10-L scenarios takes approximately 7 and 6 seconds, respectively; the ABM takes approximately 193 and 78 seconds, respectively. In the ABM, for scenarios 2-H and 10-L, we have 59535 and 24862 agents/cells, respectively. In our cgABM calibration, the computational time was between 3 to 12 hours, with 2-H and 10-L taking 4 hours 47 minutes and 31 seconds and 3 hours 57 minutes and 30 seconds, respectively. If we assume that the ABM calibration time would be proportional to the difference between a single run of the cgABM and the ABM, the ABM calibration of scenarios 2-H and 10-L would take 5 days 12 hours 7 minutes and 14 seconds and 2 days 3 hours 27 minutes and 30 seconds, respectively. Thus, while the forward model itself is fast even for the full ABM (less than 4 minutes), the coarse-grain version of the model is required to make calibration practical as it requires many forward runs of the model.

### Multi-scenario calibration and prediction

In the previous section, we made use of the scenario-specific calibration to investigate the capability of the cgABM to capture the experimentally observed evolutions of live and dead cells. In this section, we study the ability of the calibrated model to *predict* the evolution of live and dead cells in a range of initial conditions. To this end, we implement two parameter identification strategies; namely, the “multi-scenario calibration” and “leave-one-out calibration”. The purpose of multi-scenario calibration is to make use of Bayesian inference to determine one set of cgABM parameters that represent live and dead cell evolution of all scenarios with different initial tumor confluences and glucose concentrations. For the multi-scenario calibration, the reported error is the comparison among the cgABM output and each experimental scenario. In the leave-one-out approach we calibrate the cgABM parameters using the data of eight scenarios, excluding one data set (prediction scenario) from the calibration process. We then compare the calibrated cgABM simulation with measured data in the prediction scenario to investigate the model’s ability to forecast tumor responses in scenarios not included in the calibration data. Table 2 shows the error between the model output and the experimental measurements according to Eq (30). For the leave-one-out, the reported means and the 95% credible intervals in Table 2 show the discrepancies between model and data in the prediction scenarios; i.e., the data sets left out of the calibration data.

**Fig 13:**
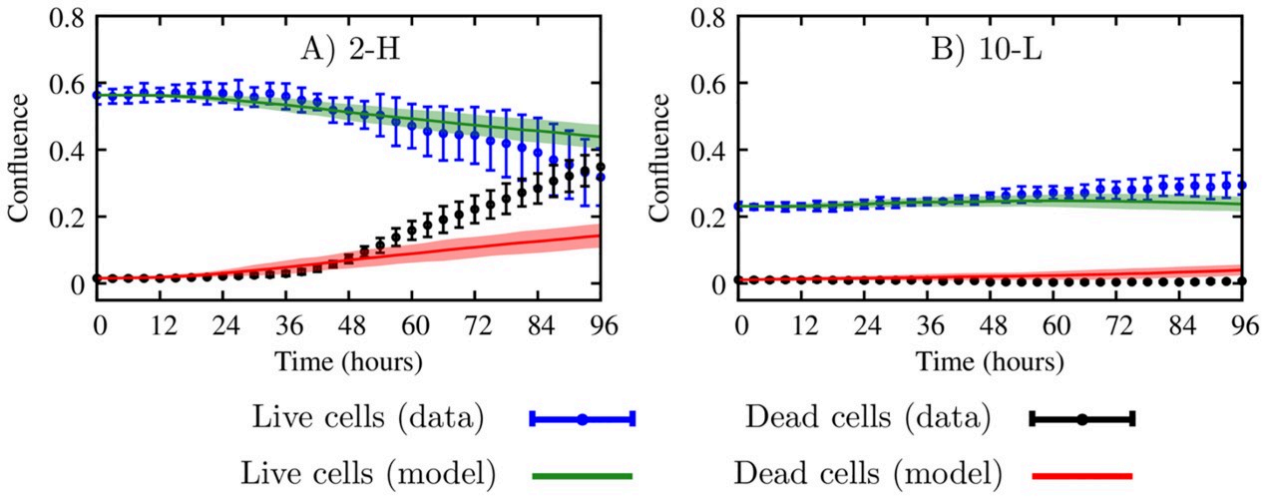
Model predictions of scenarios 2-H and 10-L. Leave-one-out prediction of scenarios 2-H (A) and 10-L (B). The cgABM was calibrated using the time-resolved microscopy data for all scenarios, excluding 2-H (panel A) and 10-L (panel B). The data mean and 95% credible interval for the live and dead cells are shown in blue and black, respectively. The mean of the simulation is represented as a solid line, and the area is the 95% credible interval for the live (green) and dead (red) cells. The average error and standard deviation for live and dead cells in scenario 2-H are 13.97 ± 3.69% and 15.80 ± 5.59%, respectively. While that for scenario 10-M are 7.61 ± 2.01% and 5.78 ± 1.13%, respectively.

Fig 13 compares the computational prediction of cgABM with the unseen experimental data for two representative cases of the leave-one-out calibration. In Fig 13A, we illustrate predicting scenario 2-H when the cgABM is calibrated with the other eight scenarios. The 2-H (high initial tumor confluence and 2 mM initial glucose) scenario was not included in the calibration process, and is the one with the highest dead cell confluence (see Fig 8) among the nine scenarios. The prediction errors of the leave-one-out calibration in Table 2 demonstrate the influence of using observational data of the 2-H scenario in informing the model parameters. That is, by excluding 2-H from the calibration data, the cgABM is unable to precisely capture the dynamics of the dead cells. Thus, this case results in an invalid cgABM for predicting the *in vitro* tumor cell behavior (if we consider the tolerance of the average error, *ε*_tol_, being 10%).

Fig 13B shows predicting scenario 10-L when the cgABM is calibrated with the other eight scenarios. The 10-L (low initial tumor confluence and 10 mM initial glucose) scenario, the confluence of the dead cells is constant over time, and the growth rate of the tumor cells is negligible (see Fig 8). From the prediction errors in Table 2, one can conclude that the 10-L scenario does not provide new information related to the tumor cells dynamics for calibrating the model parameters. That is, the other eight scenarios are sufficient to inform the dynamics of live and dead cells, resulting in a valid cgABM for computational prediction of the tumor cell behavior in a wide range of initial conditions.

**Fig 14:**
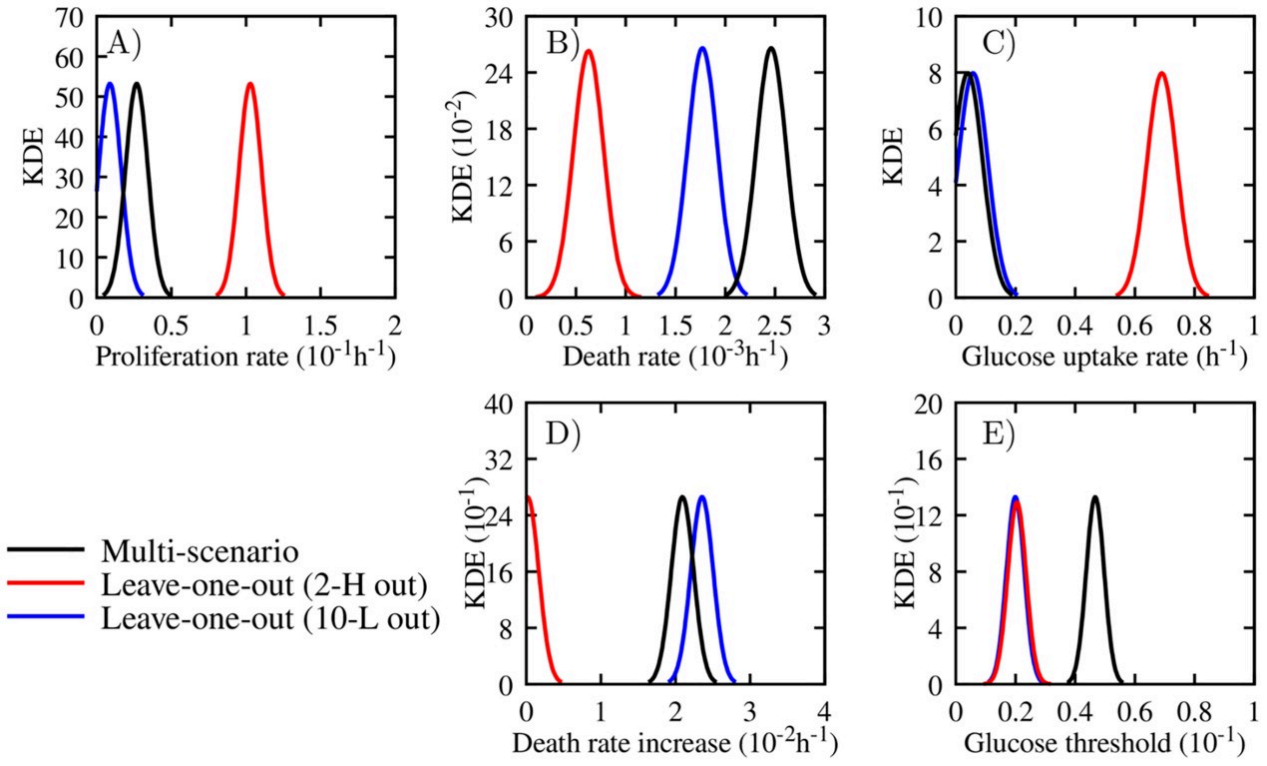
Posterior kernel density estimation (KDE) of the multi-scenario and the leave-one-out experiments. The KDE obtained during the calibration of the multi-scenario calibration *π*_post_(***θ***|**D**) (black), the high tumor initial condition and 2 mM glucose condition left out *π*_post_(***θ***|**D**_∼2*H*_) (red), and the low tumor initial condition and 10 mM glucose condition left out *π*_post_(***θ***|**D**_∼10*L*_) (blue) for the following parameters: ${\bar{\alpha }}_{P}$ (proliferation rate—panel A), ${\bar{\alpha }}_{D}$ (death rate—panel B), λ (glucose uptake rate—panel C), *γ*_*D*_ (death rate increase—panel D), and *σ*_*H*_ (glucose threshold—panel E). Leaving scenario 2-H out of the calibration causes a stronger shift in the parameter distributions than leaving out scenario 10-L. This indicates that 2-H is a scenario that cannot be represented adequately by the other experiments.

Fig 14 shows the kernel density estimate (KDE) of the parameter posteriors obtained from the multi-scenario calibration process. This figure also shows the parameter posteriors for the two representative results of the leave-one-out calibrations from the calibration data (i.e., those presented in Fig 13). Rigorous parameter estimation using different measurements must account for the data uncertainty in each training set. Characterization of the degree of confidence in the estimated parameter is critical in assessing the reliability of model prediction. The Bayesian method used in this work provides a suitable framework for quantifying the uncertainty in each measurement scenario. Comparing the parameter posteriors in Fig 14 with those obtained from scenario-specific calibration (Fig 9) indicate the posterior distributions of the multi-scenario are affected more by the data with lower uncertainty (see error bars in Fig 8). That is, poor characterization of measurement error during calibration leads to bias in model prediction.

Table 3 presents the MAP estimated of the posterior distributions from Fig 14. The MAP estimates of Table 3 demonstrate that the difference between the parameters of the calibration excluding 10-L data and the ones obtained when calibrating the nine scenarios together is smaller than the discrepancy between the posteriors of the multi-scenario *π*_post_(***θ***|**D**) and the calibration excluding 2-H *π*_post_(***θ***|**D**_∼2*H*_). These remarks confirm the importance of including the 2-H experimental data into the calibration process to adequately inform the model parameters and enhance the predictive capability of the cgABM.

**Table 3:** Maximum A Posteriori (MAP) estimates.

Parameter	Maximum A Posteriori (MAP)		
	Multi-scenario *π*_post_(*θ*|D)	Leave-one-out *π*_post_(*θ*|D_∼2*H*_)	Leave-one-out, *π*_post_(*θ*|D_∼10*L*_)
Proliferation rate (h^-1^)	2.65 × 10^−2^	1.03 × 10^−1^	9.05 × 10^−2^
Death rate (h^-1^)	2.47 × 10^−3^	6.29 × 10^−4^	1.77 × 10^−3^
Glucose uptake rate (h^-1^)	3.88 × 10^−2^	6.90 × 10^−1^	5.92 × 10^−2^
Death rate increase (h^-1^)	2.09 × 10^−2^	1.34 × 10^−4^	2.35 × 10^−2^
Glucose threshold	4.68 × 10^−2^	2.04 × 10^−2^	2.00 × 10^−2^

MAP estimates of the parameter posteriors for the multi-scenario calibration and the two cases (excluding scenarios 2-H and 10-L) of the leave-one-out calibration (see Fig 14).

## Discussion

We have presented a hybrid stochastic agent-based model to simulate the interaction among tumor cells and glucose consumption. In this model, the tumor cell movement, growth, and phenotypic transitions are represented by a discrete cellular-scale model, while a continuum tissue-scale model governs the glucose evolution. In our model, tumor cell proliferation and death are stochastic events proportional to the available glucose. The tumor cell proliferation reduces as the glucose decreases, and the chances of cell death increase if the glucose is below a threshold.

We investigated the validity of the hybrid multiscale ABM in predicting *in vitro* experimental data of human breast carcinoma cells in nine scenarios, with combinations of different initial glucose concentrations and tumor confluence. To this end, we addressed several challenges in model calibration and predictive hybrid multiscale ABMs. To overcome the high computational cost of the ABM, we coarse-grained the discrete model such that one agent represents a cluster of cells. By controlling the coarse-graining error below 5%, the developed cgABM enables simulating the entire domain of the *in vitro* experiment plate in a realistic time scale. The higher variations of the hybrid cgABM with a higher coarse-graining degree indicate that although coarse-graining of ABMs leads to a computationally tractable model, parameter inference of cgABMs demands methods that cope with significant stochasticity of the model. We also note that studying the predictive capabilities of cgABMs is critically needed for unrestricted use of these models in the cancer community, given the current limitations of computing power.

The time-dependent variance-based sensitivity analysis method was employed to identify how each parameter contributes to the model outputs (live and dead cell confluences) during the system development. The results of the sensitivity analysis showed that the most influential parameter in the multiscale cgABM is the death rate increase due to the lack of glucose (*γ*_*D*_), followed by the glucose threshold (*σ*_*H*_), and the glucose consumption rate (λ). The total effect sensitivity indices of the proliferation and death intensity reach values above 0.2 during the tumor development. The sensitivity analysis results allow us to determine which parameters must be accurately calibrated to represent the data. This analysis also helps design future experiments that can help to improve the calibration of model parameters. Two examples of experimental measurements that would significantly improve the cgABM calibration and its computational prediction are 1) temporal measurement of glucose concentration; 2) the use of hypoxia markers to track hypoxic cell confluence. Such experimental measurements would enable a more rigorous investigation of the glucose consumption rate (λ) and the glucose threshold (*σ*_*H*_) that increases death and affects proliferation.

Directed by the sensitivity analysis, a series of statistical calibrations of the cgABM using the *in vitro* experimental data were conducted. Although the data employed for calibration is limited to the temporal domain (i.e., we do not make use of the spatial information), the new cgABM parameters can be well-informed if the uncertainty is precisely characterized during the calibration process. A generalized likelihood function was proposed within the Bayesian inference to account for the intrinsic stochasticity of the cgABM in the statistical inverse problem. To perform the computationally expensive sampling algorithms for the Bayesian inferences of the stochastic cgABM, we implemented a computational infrastructure with efficient use of high-performance computing resources. We presented three model identification strategies. First, the results of scenario-specific calibration of cgABM to the time-resolved microscopy measurements indicate that the developed hybrid multiscale model can predict the experimentally observed responses of human breast carcinoma cells in a wide range of initial glucose concentrations and tumor confluences. Comparing the calibrated parameters for all nine scenarios shows that the cgABM can recapitulate the main features and the underlying tumor cell development mechanisms, with an average error of less than 8%. These processes include cell proliferation and mitosis in favorable glucose environments, as well as cell death due to lack of glucose and through apoptosis. Furthermore, the Bayesian inference method used for parameter calibration allows for assessing the associated uncertainties in model parameters while handling the inherent stochasticity of the cgABM. Second, a multi-scenario calibration was performed to identify the cgABM parameters from the in vitro data of all nine experimental data sets. The model calibration using multiple data sets with different data noise levels indicates the requirement of rigorous characterization of uncertainty in model parameters. Thus, employing Bayesian inference for statistical calibration of the ABMs is essential for characterizing the level of confidence in their computational prediction. Third, we designed an extensive calibration process, based on the leave-one-out approach, to challenge the cgABM by testing its validity in predicting unseen data. To this end, the cgABM is calibrated using the data of eight scenarios, excluding one data set (prediction scenario) from the calibration process. The results of leave-one-out calibration indicate the developed cgABM can predict the *in vitro* experiments with an error ranging from 5% to 21%. In particular, if the calibration data include the scenarios with distinct dead cell dynamics, the calibrated cgABM can precisely capture the accumulation of the dead cells, and it is a valid model for predicting the *in vitro* tumor cell behavior according to our validation criteria.

Recently, there are similar efforts in the literature on the calibration of agent-based models to tumor cells [26, 75, 76]. Jagiella *et al*. [75] calibrated a single cell-based mathematical model for multi-cellular tumor spheroids using non-small cell lung cancer (NSCLC) data. Similar to our model, the phenotypic states in their model allowed for proliferative, quiescent, and dead agents. However, the main difference between their model and the one presented here, besides their model being 3D and lattice-based, is the number of environmental factors considered in their model. In [75], the glucose, oxygen, lactate, extracellular matrix, and waste material released by dying cells are also modeled. According to the authors, this was the simplest model to capture their data, consisting of the growth kinetics and the corresponding spatial staining patterns for nuclei, different cell states, and cell environments. Although our model is simpler compared to [75], it is able to simulate our experimental observations accurately. A similar result between our model and the on in [75] is that apoptosis does not play an essential role in dead cell dynamics. As demonstrated by our sensitivity analysis, the effects of limited glucose, which translates into an increase in cell mortality (*γ*_*D*_), is the most important factor to determine the dynamics of dead cells. In [76], a hybrid ABM is developed to model the growth of multi-cellular tumor spheroids. The possible cell phenotypes are quiescent, proliferative, hypoxic, dead, and necrotic (dead cells undergoing cytolysis). The environmental factors modeled are oxygen, glucose, and the concentration of a hypoxia-activated prodrug (SN30000). To capture the radiation’s effects, the linear-quadratic model was employed by [77]. The data used for the calibration comes from *in vitro* experiments with human colon cancer cells treated with radiation, in a range of dose rates (0.2–1 Gy/min) and SN30000. Among the measurements used are the temporal changes of the spheroid diameter, number of cells, cell viability, hypoxic fraction, and S-phase cell fraction. In their model, apoptosis is not considered, with the death of the tumor cells being due to oxygen or glucose deprivation and the treatment effects. This assumption is aligned with the results found in our calibration, where *γ*_*D*_ is at least ten times higher than apoptosis in every scenario. One main difference between our model and the ones in [75, 76], is that we developed a lattice-free model, which is generally more computationally expensive (considering the same number of model constituents). The methodology employed here for model coarsening allows us to perform statistical calibration considering the model stochasticity.

While the proposed hybrid two-scale ABM can represent the tumor cell responses observed in the experimental measurements, there remain several areas that can be further developed in future studies. In the current effort, the spatial distribution of the tumor cells was not taken into account during the model calibration (i.e., only the confluence data was used in the model calibration). Thus, we are not considering the mobility of the tumor, as previously done in [3]. This effect would be necessary to characterize, for example, angiogenesis [28]. The position of the tumor cells influences the growth pattern of the new vasculature. Another direction for investigation is to calibrate the ABM using 3D tumor platforms [78–81]. However, a limiting factor in our 3D simulations would be the computational cost characteristic of discrete models with multiple agents. The 2D cgABM simulation simulates the observational data within a 5% error compared to the one cell per agent ABM. However, we expect the coarse-graining error would be more substantial in 3D simulations. Also, we showed that the cgABM was able to capture the evolution of human breast carcinoma cells, in three different initial confluences and three different glucose availability, with an average error of less than 8%. One aspect that could improve the simulation is a better characterization of the initial distribution of the cell cycle within the tumor cells. The current model considers all cells initially in a quiescent state. Additionally, labeling the cell phenotypes in the *in vitro* experiments, see, e.g., [82] could provide better observational data to portray the initial conditions. Currently, our proliferation dynamics (as determined by Eq (14)) are only able to capture the tumor dynamics in glucose concentrations below 10 mM (as the 1 in Eq (14) represents the maximum, normalized, glucose concentration used experimentally). These scenarios with 2 and 5 mM initial glucose are compared to the scenario 10 mM.

In our current modeling approach, the goal is to develop the most parsimonious model that is capable of describing the data with the least number of free parameters. Thus, we assume that glucose is the only factor that affects cell death and proliferation (the stochastic events guided by Eqs (11) and (12), respectively). However, other mechanisms such as mechanical and chemical interactions between cells and the extracellular environment also (of course) have an essential role in cellular dynamics. The variation in parameters can imply that there can be other factors governing the dynamics that are not captured by these parameters (due to the modeling assumptions and simplifications) and therefore affecting their values. If we were to extend the model to account for these phenomena, we would almost certainly require more measurements of different processes to be able to calibrate the model accurately. For example, in the current model, high initial glucose concentration is not necessarily reflected by a higher proliferation rate. This result implies that there are other factors that can limit the proliferation rate besides the availability of glucose—factors that are currently not accounted for in our model. Regarding the glucose uptake rate, the value decreases as the glucose concentration increases due to the lack of dead cells in scenarios with high initial glucose concentration. In our model, the death probability is inversely proportional to the glucose concentration (i.e., in an environment with high glucose concentration, the cells have a low death probability of death). Thus, to avoid the increase in the number of dead cells, the model calibration converges to a lower glucose uptake rate to keep the glucose concentration high. We believe that there could be an alternative relationship between the death rate and glucose concentration. However, no data on glucose concentration as a function of time were collected during the experiment. Thus, with the currently available data, we were not able to constrain the values of the glucose over time. However, the pattern/relationship found in our model parameters, such as the variation in proliferation rate as the initial conditions change, can be used to develop a “look-up” table. Such an approach helps to “personalize” each prediction as these model parameters are determined by the initial condition of each individual experiment. Furthermore, the initial conditions are (by definition) frequently known (or, at least, bounded) at the beginning of the experiment, providing a practical way to constrain model predictions. Finally, only temporal observational data is employed in this study for model calibration as a first step in developing predictive ABMs with quantified uncertainty. Future efforts will investigate the simultaneous integration of spatial and temporal experimental data within the cgABM framework to enable the practical application of hybrid multiscale models in cancer.

## Conclusions

We have developed a new hybrid, two-scale, stochastic agent-based model of tumor cell dynamics and investigated the model’s ability to simulate and predict *in vitro* experimental observations of live and dead cell numbers over time, given the initial conditions. The first major contribution of this work is the development of a coarse-grained ABM (cgABM) with a reduced parameter space and significantly lower computational costs. The second major contribution is the rigorous characterization of uncertainty in the prediction provided by the cgABM using time-dependent sensitivity analysis and Bayesian inference that handles the stochasticity of the model. The cgABM we developed reduces the computational time of ABM simulations by factors of 13 to 28 with less than 3% average error compared to the ABM. This study also indicates that our multiscale ABM can reliably predict the temporal evolution of cancer cells subjected to a wide range of initial conditions. The advances made in this manuscript to develop a reduced-order ABM while addressing model stochasticity *via* Bayesian parameter inference represents a significant step towards applying predictive, spatially, and temporally resolved ABMs in cancer a practical reality.

## Supporting information

S1 FigLow confluence sensitivity analysis.Sensitivity analysis of the proliferation rate (${\bar{\alpha }}_{P}$), death rate (${\bar{\alpha }}_{D}$), glucose diffusion (*D*), glucose uptake (λ), death rate increase due to lack of glucose (*γ*_*D*_), transition contrast (*k*), and glucose threshold (*σ*_*H*_) for live (top row) and dead (bottom row) cell phenotypes seeded with low confluence. Panels A-F show the total effect index over time with Panels A, B, and C depicting live tumor cells, while Panels D, E, and F depict the dead tumor cells. The importance of the parameters is studied for three initial glucose concentrations: 2 mM (Panels A and D), 5 mM (Panels B and E), and 10 mM (Panels C and F). The glucose diffusion and the smooth transition constant have limited influence on the quantities of interest during the complete simulation (i.e., large changes in these parameters would yield small changes in tumor composition). Apart from these two parameters, the total effect index for every parameter is greater than 0.2 during the 96 hours simulated.(TIF)

S2 FigHigh confluence sensitivity analysis.Sensitivity analysis of the proliferation rate (${\bar{\alpha }}_{P}$), death rate (${\bar{\alpha }}_{D}$), glucose diffusion (*D*), glucose uptake (λ), death rate increase due to lack of glucose (*γ*_*D*_), transition contrast (*k*), and glucose threshold (*σ*_*H*_) for live (top row) and dead (bottom row) cell phenotypes seeded with high confluence. Panels A-F show the total effect index over time with Panels A, B, and C depicting live tumor cells, while Panels D, E, and F depict the dead tumor cells. The importance of the parameters is studied for three initial glucose concentrations: 2 mM (Panels A and D), 5 mM (Panels B and E), and 10 mM (Panels C and F). The glucose diffusion and the smooth transition constant have limited influence on the quantities of interest during the complete simulation (i.e., large changes in these parameters would yield small changes in tumor composition). Apart from these two parameters, the total effect index for every parameter is greater than 0.2 during the 96 hours simulated.(TIF)

S1 Appendix Parameter identifiability analysis. Before conducting the Bayesian calibration of the cgABM against the *in vitro* experimental data, we perform a parameter identifiability analyses. To this end, we make use of the cgABM with a set of “true’’ parameter values to generate *in silico* data. We then calibrate the cgABM parameters against the *in silico* data using Bayesian inference. To check the parameter identifiability, we compare the true parameter values with the MAP estimates of the calibration posteriors.
(PDF)

S2 Appendix Bayesian calibration computation. For all calibrations, the Bayesian inference is conducted using 16 chains within the adaptive multi-level Monte Carlo algorithm, along with computing the means of *N*_*r*_ = 17 realizations of the model per sample. As each forward model is computed in serial, the total number of processors per simulation is the number of chains times the number of realizations (i.e., 272 processors in the scenario-specific calibration). For the multi-scenario and the leave-one-out calibration, we multiply this number of processors by the number of scenarios used in the calibration. In this appendix, we present the computational time for all the calibration experiments presented in this work.
(PDF)
