# Supplementary figures and images for: Bayesian calibration of a stochastic, multiscale agent-based model for predicting in vitro tumor growth
Source: PLoS Comput Biol. 2021 Nov 29;17(11):e1008845. doi: 10.1371/journal.pcbi.1008845 (PMC8659698; doi:10.1371/journal.pcbi.1008845)

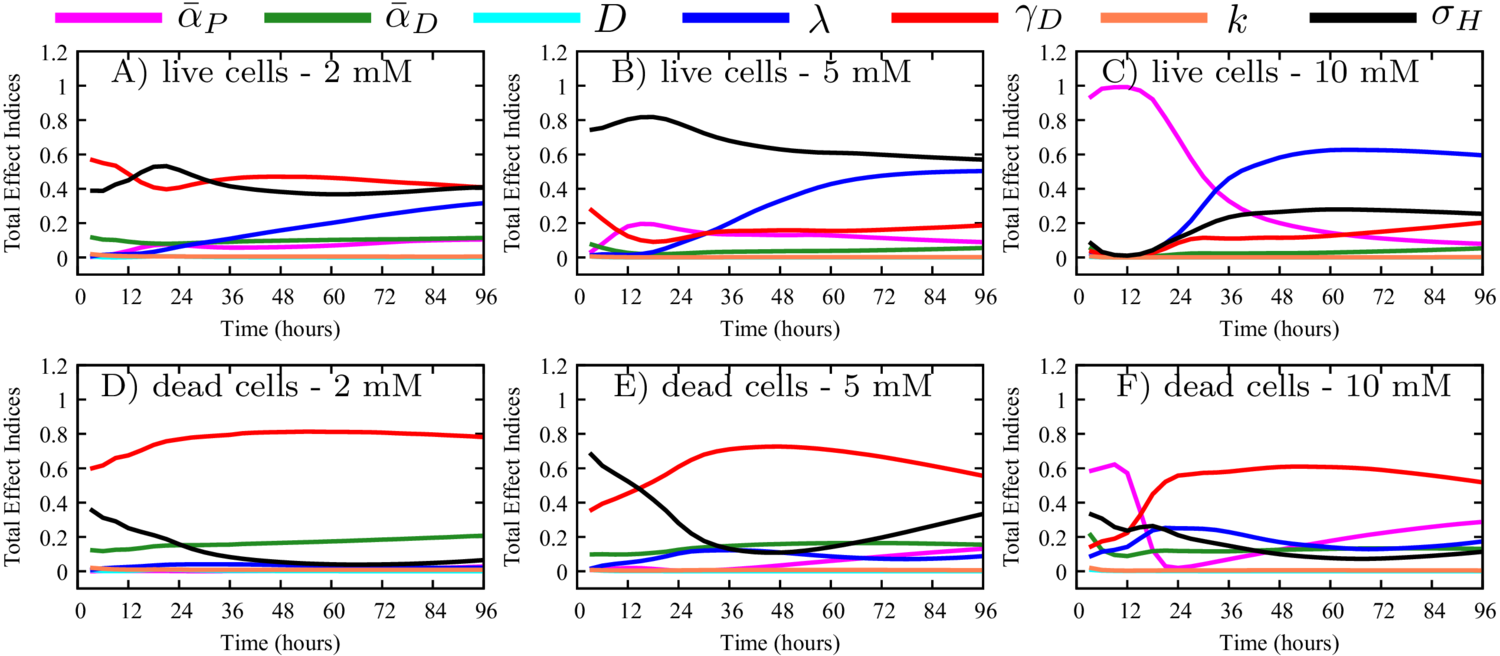

Supplement: S1 Fig — Sensitivity analysis of the proliferation rate (α¯P), death rate (α¯D), glucose diffusion (D), glucose uptake (λ), death rate increase due to lack of glucose (γD), transition contrast (k), and glucose threshold (σH) for live (top row) and dead (bottom row) cell phenotypes seeded with low confluence. Panels A-F show the total effect index over time with Panels A, B, and C depicting live tumor cells, while Panels D, E, and F depict the dead tumor cells. The importance of the parameters is studied for three initial glucose concentrations: 2 mM (Panels A and D), 5 mM (Panels B and E), and 10 mM (Panels C and F). The glucose diffusion and the smooth transition constant have limited influence on the quantities of interest during the complete simulation (i.e., large changes in these parameters would yield small changes in tumor composition). Apart from these two parameters, the total effect index for every parameter is greater than 0.2 during the 96 hours simulated. (TIF) [file pcbi.1008845.s001.tif]

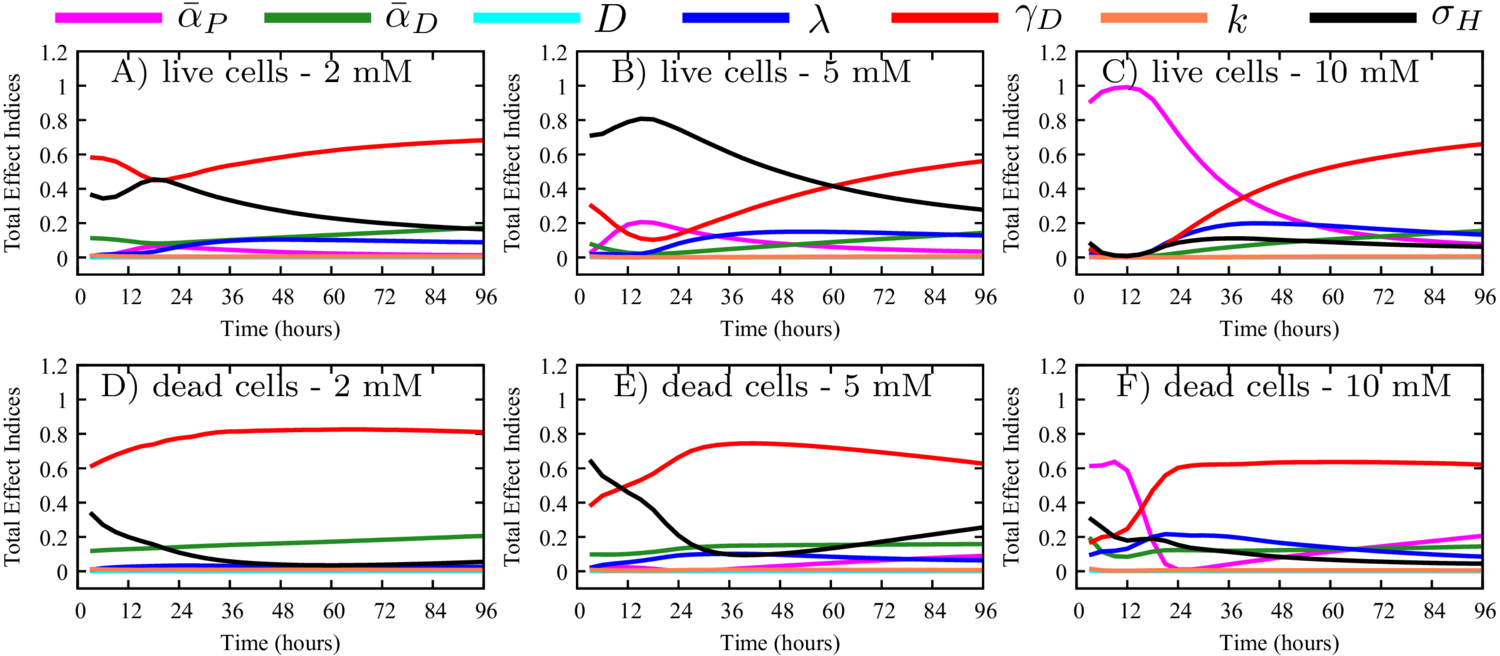

Supplement: S2 Fig — Sensitivity analysis of the proliferation rate (α¯P), death rate (α¯D), glucose diffusion (D), glucose uptake (λ), death rate increase due to lack of glucose (γD), transition contrast (k), and glucose threshold (σH) for live (top row) and dead (bottom row) cell phenotypes seeded with high confluence. Panels A-F show the total effect index over time with Panels A, B, and C depicting live tumor cells, while Panels D, E, and F depict the dead tumor cells. The importance of the parameters is studied for three initial glucose concentrations: 2 mM (Panels A and D), 5 mM (Panels B and E), and 10 mM (Panels C and F). The glucose diffusion and the smooth transition constant have limited influence on the quantities of interest during the complete simulation (i.e., large changes in these parameters would yield small changes in tumor composition). Apart from these two parameters, the total effect index for every parameter is greater than 0.2 during the 96 hours simulated. (TIF) [file pcbi.1008845.s002.tif]
