# Supplementary material for: Bayesian calibration of a stochastic, multiscale agent-based model for predicting in vitro tumor growth
Source: PLoS Comput Biol. 2021 Nov 29;17(11):e1008845. doi: 10.1371/journal.pcbi.1008845 (PMC8659698; doi:10.1371/journal.pcbi.1008845)
Supplement: S1 Appendix — Before conducting the Bayesian calibration of the cgABM against the in vitro experimental data, we perform a parameter identifiability analyses. To this end, we make use of the cgABM with a set of “true’’ parameter values to generate in silico data. We then calibrate the cgABM parameters against the in silico data using Bayesian inference. To check the parameter identifiability, we compare the true parameter values with the MAP estimates of the calibration posteriors. (PDF) [file pcbi.1008845.s003.pdf]

S1 Appendix. Parameter Identifiability Analysis.

As the hybrid multiscale ABM is quite complete with many model parameters, it is natural to determine (before model calibration against experimental observations) if it is feasible to infer the parameters of the model from data. Unidentifiable parameters are the model parameters that cannot be learned from a set of "perfect" data resulting in the same model predictions for more than one value of the parameters [1]. In a Bayesian setting, different combinations of unidentifiable parameters lead to the same likelihood. Additionally, due to strong correlations among the posterior probability distribution of the parameters, the existence of unidentifiable model parameters slows down the convergence rate of MCMC algorithms [2].

For these reasons, before conducting the Bayesian calibration of the cgABM against the *in vitro* experimental data, we perform a parameter identifiability analyses. To this end, we make use of the cgABM with a set of "true" parameter values to generate *in silico* data. We then calibrate the cgABM parameters against the *in silico* data using Bayesian inference. To check the parameter identifiability, we compare the true parameter values with the MAP estimates of the calibration posteriors.

Table A shows the true values of the cgABM parameters used to generate the set of *in silico* data. The true parameters are chosen such that the data show the initial increase in live cell confluence, followed by decrease of live cell confluence due to the lack of glucose in the microenvironment, and, consequentially, an increase in dead cells. The domain size, degree of coarse-graining, and other simulation features are the same as those used for the sensitivity analyses and calibration in the main manuscript. The MAP estimates of the calibrated parameters inferred from the data are shown in Table A. We observe that the inferred parameters are close to their true values with a maximum discrepancy of 25% in the glucose threshold. Fig A presents the *in silico* data and the cgABM outputs using the calibration posterior. The comparison between data and model in this figure indicates that the model can capture the main features of the data with an average error below 0.21%.

Table A. Parameter identifiability.

Parameter	True value	Maximum A Posteriori
Proliferation intensity	4.90e-02	4.96e-02
Death intensity	4.10e-04	5.08e-04
glucose uptake rate	4.80e-02	5.73e-02
Death rate increase	2.40e-02	2.83e-02
glucose threshold	5.40e-02	4.06e-02

True parameters value used to generate the *in silico* data and the values of MAP estimates obtained from calibration posteriors.

The results of the parameter identifiability analysis in Table A and Fig A indicate that the cgABM calibration parameters are identifiable. They also verify our Bayesian calibration approach and computational infrastructure to be used for calibrating the cgABM against *in vitro* experiments.

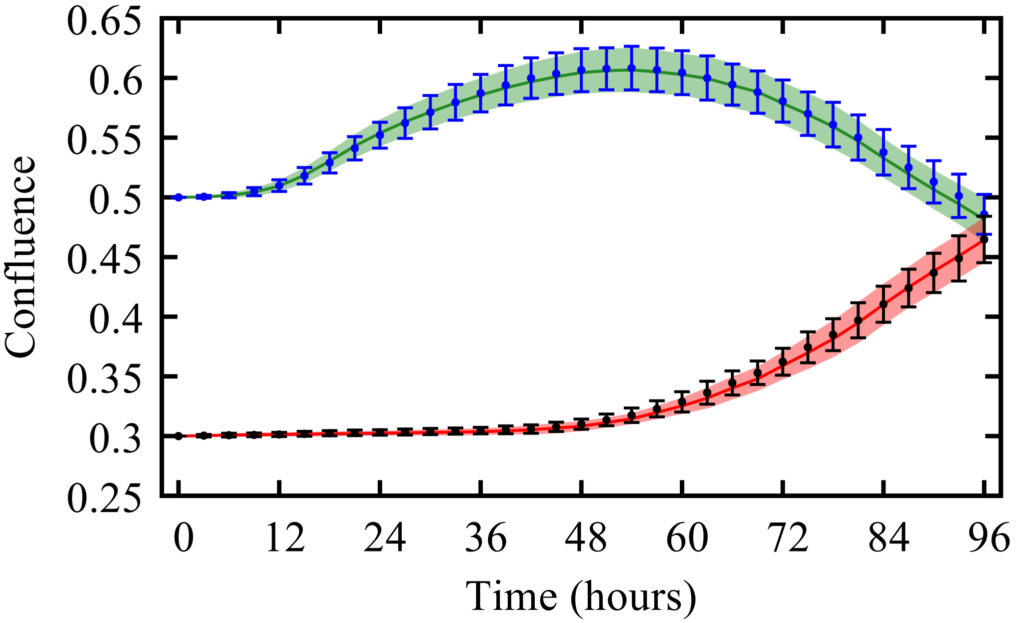

Figure A. Spatiotemporal evolution of the *in silico* data and the calibrated model. Comparison between *in silico* data (points with 95% error bars) and model calibration (mean as a continuous line and 95% confidence interval as shadow area). The mean relative error for the living (blue and green) and dead (black and red) cells were $0.22 \pm 0.06\%$ and $0.19 \pm 0.05\%$, respectively.

References

1. Rateitschak K, Winter F, Lange F, Jaster R, Wolkenhauer O. Parameter identifiability and sensitivity analysis predict targets for enhancement of STAT1 activity in pancreatic cancer and stellate cells. PLoS computational biology. 2012;8(12).

2. Rannala B. Identifiability of parameters in MCMC Bayesian inference of phylogeny. Systematic Biology. 2002;51(5):754–760.
