# Supplementary material for: Bayesian calibration of a stochastic, multiscale agent-based model for predicting in vitro tumor growth
Source: PLoS Comput Biol. 2021 Nov 29;17(11):e1008845. doi: 10.1371/journal.pcbi.1008845 (PMC8659698; doi:10.1371/journal.pcbi.1008845)
Supplement: S2 Appendix — For all calibrations, the Bayesian inference is conducted using 16 chains within the adaptive multi-level Monte Carlo algorithm, along with computing the means of Nr = 17 realizations of the model per sample. As each forward model is computed in serial, the total number of processors per simulation is the number of chains times the number of realizations (i.e., 272 processors in the scenario-specific calibration). For the multi-scenario and the leave-one-out calibration, we multiply this number of processors by the number of scenarios used in the calibration. In this appendix, we present the computational time for all the calibration experiments presented in this work. (PDF) [file pcbi.1008845.s004.pdf]

S2 Appendix. Bayesian calibration computation.

For all calibrations, the Bayesian inference is conducted using 16 chains within the adaptive multi-level Monte Carlo algorithm, along with computing the means of $N_r = 17$ realizations of the model per sample. As each forward model is computed in serial, the total number of processors per simulation is the number of chains times the number of realizations (i.e., 272 processors in the scenario-specific calibration). For the multi-scenario and the leave-one-out calibration, we multiply this number of processors by the number of scenarios used in the calibration. In Table A, we present the computational time for all the calibration experiments presented in this work.

Table A. Model parameters.

Scenario	Scenario-specific	Leave-one-out
2-L	04 h 33 min and 35 s	26 h 38 min and 45 seconds
2-M	04 h 46 min and 01 s	33 h 59 min and 18 seconds
2-H	04 h 47 min and 31 s	28 h 00 min and 34 seconds
5-L	03 h 38 min and 54 s	24 h 09 min and 30 seconds
5-M	03 h 53 min and 50 s	16 h 57 min and 24 seconds
5-H	05 h 48 min and 24 s	34 h 29 min and 08 seconds
10-L	03 h 57 min and 30 s	37 h 40 min and 34 seconds
10-M	05 h 54 min and 16 s	15 h 50 min and 40 seconds
10-H	12 h 05 min and 05 s	13 h 40 min and 10 seconds

Computational time for the scenario-specific and the leave-one-out calibrations. The middle and right columns indicate the scenario-specific calibrated and the scenario left out during the calibration for the leave-one-out experiments, respectively. The computational time for the multi-scenario calibration was 45 h 23 min and 25 s.
